# Positive selection for unpreferred codon usage in eukaryotic genomes

**DOI:** 10.1186/1471-2148-7-119

**Published:** 2007-07-18

**Authors:** Daniel E Neafsey, James E Galagan

**Affiliations:** 1 Microbial Analysis Group, Broad Institute of MIT and Harvard, 7 Cambridge Center, Cambridge, MA 02142, USA

## Abstract

**Background:**

Natural selection has traditionally been understood as a force responsible for pushing genes to states of higher translational efficiency, whereas lower translational efficiency has been explained by neutral mutation and genetic drift. We looked for evidence of directional selection resulting in increased unpreferred codon usage (and presumably reduced translational efficiency) in three divergent clusters of eukaryotic genomes using a simple optimal-codon-based metric (K_p_/K_u_).

**Results:**

Here we show that for some genes natural selection is indeed responsible for causing accelerated unpreferred codon substitution, and document the scope of this selection. In *Cryptococcus *and to a lesser extent *Drosophila*, we find many genes showing a statistically significant signal of selection for unpreferred codon usage in one or more lineages. We did not find evidence for this type of selection in *Saccharomyces*. The signal of positive selection observed from unpreferred synonymous codon substitutions is coincident in *Cryptococcus *and *Drosophila *with the distribution of upstream open reading frames (uORFs), another genic feature known to reduce translational efficiency. Functional enrichment analysis of genes exhibiting low K_p_/K_u _ratios reveals that genes in regulatory roles are particularly subject to this type of selection.

**Conclusion:**

Through genome-wide scans, we find recent selection for unpreferred codon usage at approximately 1% of genetic loci in a *Cryptococcus *and several genes in *Drosophila*. Unpreferred codons can impede translation efficiency, and we find that genes with translation-impeding uORFs are enriched for this selection signal. We find that regulatory genes are particularly likely to be subject to selection for unpreferred codon usage. Given that expression noise can propagate through regulatory cascades, and that low translational efficiency can reduce expression noise, this finding supports the hypothesis that translational efficiency may be suppressed in some cases to reduce stochastic noise in gene expression.

## Background

It is generally accepted that natural selection operates to increase translational efficiency in the genomes of unicellular as well as some multicellular organisms [1-4]. The observation that codons translated by common tRNAs are used more frequently than synonymous codons translated by rare tRNAs, and that this usage bias strengthens with gene expression level, is interpreted as a signal of selection to increase translation rate [5] and/or accuracy[6]. The observation that codons translated by rare tRNAs can significantly decrease translation rate [7,8] has likewise motivated many reports claiming evidence for the downward modulation of expression level through the use of inefficiently translated codons [9-13].

Though this analogous argument for down-regulation of expression through codon usage, known as the 'expression-regulation theory,' has a symmetric appeal, it has been strongly and repeatedly challenged [9,14,15]. Among the principal objections to the theory are simple pragmatism; because the process of transcription consumes cellular resources and energy, it would therefore be natural to assume that most constitutive (non-regulatory) constraints on gene expression levels would be imposed before, rather than after transcription. Indeed, it has even been suggested that such constraints would be more easy to evolve at the transcriptional rather than the translational level [14]. Further, it was assumed that synonymous substitutions creating inefficiently translated codons would have no perceptible phenotypic effect except in very highly expressed genes, making such mutations effectively invisible to natural selection [9,14]. Many early studies in favor of the expression-regulation theory also failed to document a significant enrichment of translationally inefficient codons in genes thought to be subject to translational repression [14], leading to the 'selection-mutation-drift theory' that weak codon bias results from an absence of selection for translational efficiency, rather than from selection in the opposite direction.

Recently, however, positive selection for inefficiently translated codons has been reported for several exons of a gene in the fruitfly *Drosophila melanogaster *[16,17], and a deficit of translationally efficient codons has been detected in some human genes [18], suggesting that some cases of inefficient translation may indeed be an evolutionarily deliberate strategy. A larger than expected *in vivo *phenotypic effect of translationally inefficient codon substitutions has also been observed at the *Adh *locus in *D. melanogaster *[8], suggesting that such mutations might be more evolutionarily labile than previously believed.

Motivated by these findings, we conducted a genome-wide scan for selection for inefficient translation in two widely divergent fungal lineages and *Drosophila*, using clusters of three to four closely related species from each lineage to observe and root recent synonymous substitutions. We use the rate of unpreferred codon substitution as a measure of selection for translational inefficiency. While this signal may also be interpretable as a measure of selection for translational inaccuracy, we know of no hypotheses predicting such selection. Further, we find that in *Cryptococcus *and *Drosophila*, the signal of positive selection observed from synonymous codon substitutions in certain genes is concordant with the distribution and conservation of upstream open reading frames (uORFs), another genic feature known to reduce translational efficiency. We find that natural selection does in fact operate at many loci across the genome in *Cryptococcus *and several loci in *Drosophila *to reduce preferred codon usage and presumably translational efficiency, but we fail to find such a signal in *Saccharomyces*. Based on functional enrichment analysis of genes showing accelerated rates of mutation towards translationally inefficient codons, we suggest this selection may be acting to minimize stochastic noise in gene expression.

## Results

### Synonymous Codon analysis

To perform genome-wide scans for selection for inefficient translation, we employed a straightforward metric that detects recent mutation-selection disequilibrium for codon usage, using partitioned counts of synonymous sites and substitutions as described by Bauer Dumont et al. [16]. To calculate this metric, synonymous codons were assigned "preferred," "unpreferred," or "equal" status according to whether their usage differed significantly between gene sets exhibiting high or low overall codon bias in each genome (Methods; Additional Files 1, 2, 3). We then classified synonymous substitutions in aligned orthologous genes within each genus as preferred or unpreferred according to the status of the ancestral and derived codons [19], using a maximum likelihood approach to infer ancestral states (Methods). For example, an unpreferred codon that changes into a preferred synonymous codon would be classified as a preferred synonymous substitution, and the opposite directionality of change would be classified as an unpreferred synonymous substitution. Counts of synonymous substitutions were corrected for multiple substitutions [20].

To create normalized rates of synonymous substitution, we divided each substitution count by the number of ancestral synonymous 'sites', or opportunities for mutation available for each class of substitution. Note that on average, preferred codons will tend to exhibit more unpreferred sites, or opportunities for unpreffered changes, than unpreferred codons, and likewise unpreferred codons will exhibit more preferred sites than preferred codons. Normalizing the counts of preferred and unpreferred substitutions by the number of ancestral preferred and unpreferred sites, respectively, therefore allows one to make fair comparisons of the relative rate of preferred to unpreferred mutations among genes exhibiting differing degrees of ancestral codon usage bias. We define K_p _as (# of preferred synonymous substitutions)/(# preferred ancestral sites) and K_u _as (# of unpreferred synonymous substitutions)/(# of unpreferred ancestral sites). Taking the quotient of K_p _and K_u _(K_p_/K_u_) then yields a metric for measuring selection on synonymous codon usage across lineages. This metric is analogous to the K_a_/K_s _statistic for measuring nonsynonymous rates of change, but differs in that it is sensitive only to changes in the selection regime in one or more lineages and not a constant, equilibrium level of selection. K_p_/K_u _is thus expected to be equal to 1 under both neutral conditions and selection-mutation equilibrium. A K_p_/K_u _ratio significantly less than 1 reflects accelerated unpreferred substitution and presumably new or intensified selection for reduced translation rate, whereas a K_p_/K_u _ratio significantly greater than 1 reflects selection for accelerated preferred substitution and presumably increased translational inefficiency. See the appendix for a more detailed examination and example application of the K_p_/K_u _metric.

**Figure 1:**
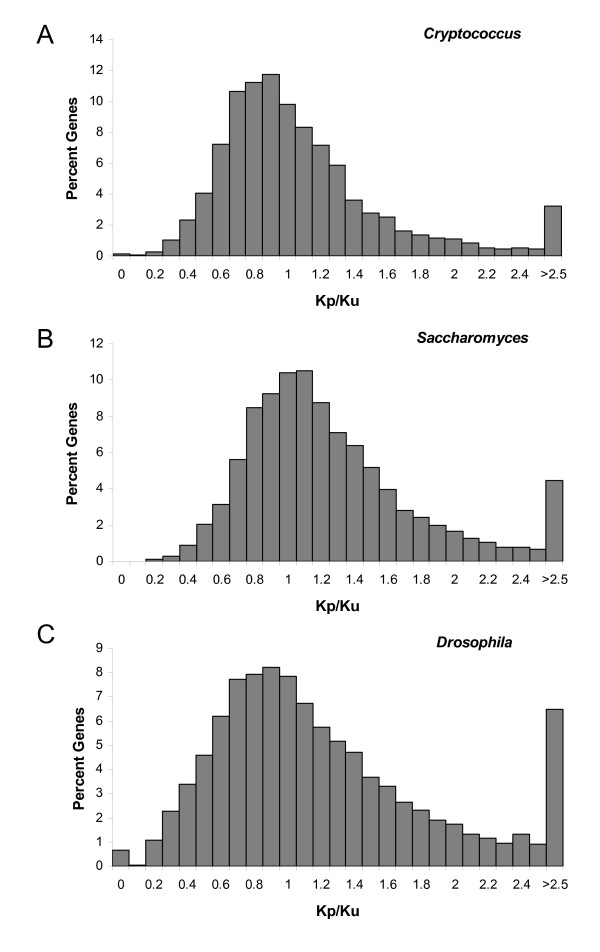
Distribution of K_p_/K_u _ratios from genes exhibiting at least 10 synonymous substitutions in (**A**) *Cryptococcus *(n = 4,993 genes; mean = 1.06; st. dev. = 0.70), (**B**) *Saccharomyces *(n = 4,878 genes; mean = 1.21; st. dev. = 0.90), and (**C**) *Drosophila *(n = 4,800 genes; mean = 1.27; st. dev. = 0.93).

We calculated the K_p_/K_u _metric for 5,450 *Cryptococcus *genes, 5,921 *Drosophila *genes, and 5,158 *Saccharomyces *genes (Figure 1). Gene set sizes were determined by the number of all-way reciprocal-best-BLAST hits that were obtained within each clade (Methods). *Cryptococcus *exhibited the strongest signal of accelerated unpreferred substitution. We found 125 *Cryptococcus *genes exhibiting K_p_/K_u _ratios less than 1 at a *p *value ≤ 0.01 (1-tailed Fisher's exact test; Additional File 4). We also found 69 *Drosophila *genes and 36 *Saccharomyces *genes exhibiting ratios with such low *p *values (Additional Files 5 &6). Q-value analysis [21] to account for multiple testing suggests a false discovery rate of 32% among the set of 125 *Cryptococcus *genes, 91% among the 96 *Drosophila *genes, and 100% among the 33 *Saccharomyces *genes, yielding approximately 85 genes in *Cryptococcus *and 6 genes in *Drosophila *that reflect strong selection for translational inefficiency mediated via accelerated unpreferred synonymous substitution in one or more lineages.

Analysis of substitution patterns in the 5' leader and 3' trailer sequences flanking these genes indicates the observed selection signal does not derive from selection on local nucleotide composition or biased mutation rates. In *Cryptococcus *and *Drosophila*, unpreferred codons uniformly exhibit an A or U and preferred codons uniformly exhibit a G or C in the third position within the tyrosine, histidine, glutamine, asparagine, lysine, aspartic acid, and glutamic acid two-fold degenerate synonymous codon families (Additional Files 1 &2). A lesser GC bias also exists among preferred codons in *Saccharomyces *(Additional File 3). This creates the possibility that genes with a low K_p_/K_u _ratio reflect localized selection for lower GC content or regional mutation bias rather than selection for translational inefficiency in these genera. To test for this, we compared patterns of nucleotide substitution in the 5' leader and 3' trailer sequences of two sets of genes from each taxonomic cluster: a set exhibiting the lowest observed K_p_/K_u _ratios (5^th ^percentile and below), and a set exhibiting the highest K_p_/K_u _ratios (95^th ^percentile and above). Ancestral and derived states were inferred for substitutions in these flanking regions in the same manner as for substitutions at synonymous coding sites (Methods).

In both fungal genera and in fruitfiles, the ratios of A/T-to-G/C and G/C-to-A/T substitution rates did not significantly differ between the high and low K_p_/K_u _gene sets (Table 1; χ^2 ^test; *Cryptococcus p *= 0.68; *Saccharomyces p *= 0.59; *Drosophila p *= 0.50). Thus we infer that genes exhibiting K_p_/K_u _ratios significantly less than or greater than 1 likely reflect selection on the translational properties of codons rather than local selection for nucleotide composition or a locally biased mutation profile.

**Table 1:** Mutation counts by class in the 5'leader and 3' trailer sequences of genes exhibiting high and low K_p_/K_u _ratios.

	Mutation Type			
			
	A/T->G/C	GC->A/T	Chi. Sq.	*P *value
*Cryptococcus*				
low K_p_/K_u_	319	333	0.2	0.66
high K_p_/K_u_	360	394		
*Saccharomyces*				
low K_p_/K_u_	165	128	0.29	0.59
high K_p_/K_u_	310	260		
*Drosophila*				
low K_p_/K_u_	219	269	0.46	0.5
high K_p_/K_u_	132	179		

While *Saccharomyces *yielded few genes with K_p_/K_u _ratios significantly less than 1, there remains evidence that the statistic is an estimator of selection on translational efficiency in this organism. We find a highly significant association between K_p_/K_u _ratio and empirical measurements of translational efficiency based on ribosome density on transcripts [22] (Additional File 7; Spearman's rho = 0.28; p < 0.00001).

**Figure 2:**
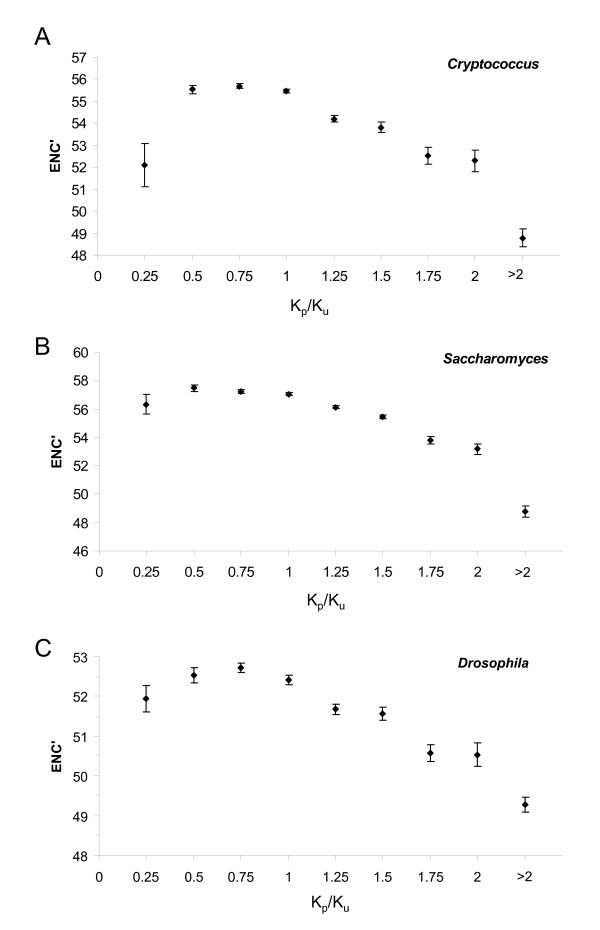
K_p_/K_u _is directly correlated with codon bias, here measured using the ENC' statistic ([49]; lower ENC' prime values indicate higher codon bias) in (**A**) *Cryptococcus*, (**B**) *Saccharomyces*, and (**C**) *Drosophila*. Error bars indicate standard error. This relationship suggests that recent selective pressures on codon usage in these groups generally reinforce historic selective pressures.

We observed that the recent selective forces on genes reflected by K_p_/K_u _are in most cases concordant with historical selection pressures on genes, as measured by codon bias. Genes exhibiting an excess of preferred synonymous substitutions (high K_p_/K_u_) tend to exhibit stronger codon bias in *Cryptococcus*, *Saccharomyces*, and *Drosophila *(Figure 2), suggesting that selection is continuing to strengthen or reinforce codon bias in those genes. However, genes exhibiting the most extreme unpreferred synonymous substitution rates (K_p_/K_u _< 0.25) demonstrate stronger average codon bias than genes exhibiting more moderate unpreferred substitution rates (K_p_/K_u _0.25–0.75), particularly in *Cryptococcus*. A similar pattern was recently observed in the human genome, where genes exhibiting the lowest incidence of optimal codons (where optimality was determined by tRNA gene counts) exhibit stronger codon bias than genes exhibiting intermediate levels of optimal codon usage [18]. In *Cryptococcus*, however, we found that genes exhibiting K_p_/K_u _ratios less than 0.25 actually exhibit a higher ratio of preferred to unpreferred codon incidence (1.47 vs. 1.40; χ^2 ^= 9.32, *p *= 0.002) than genes exhibiting more moderate K_p_/K_u _ratios (0.25–0.75), as well as a higher average count of genomic tRNAs/codon (4.66 vs. 4.36; 2-tailed *t *test, *p *= 8.4E^-11^). These results suggest that the concave shape of the curves in Figure 2 is most likely due to asymmetric variance in K_p_/K_u _for genes exhibiting strong codon bias.

### Upstream Open Reading Frame analysis

We find an association between genes with low K_p_/K_u _ratios and upstream open reading frames (uORFs). uORFs are short open reading frames located in the transcribed 5' leader sequence of genes [23]. uORFs are capable of repressing protein translation by inhibiting ribosome re-initiation at the downstream protein-coding start site, decreasing mRNA transcript stability, or encoding a cisacting peptide capable of stalling the ribosome [24]. Experimental analyses have shown that uORFs are variable in their impact on translation, but are capable of inducing up to a 20-fold reduction in translation rate [25]. We report uORFs only from genes where 5' leader length could be confidently determined by empirical data and for which we could calculate K_p_/K_u_. We found 256 uORFs at genes fitting these requirements in *Cryptococcus *strain JEC21, 642 uORFs in *D. melanogaster*, and 403 uORFs in *S. cerevisiae*. Of these total counts, 107, 417, and 102 uORFs were respectively conserved in all species of *Cryptococcus*, *Drosophila*, and *Saccharomyces *(Additional File 8). Using a Mann-Whitney *U *test, we found a statistically significant association between K_p_/K_u _and uORF presence in *Cryptococcus *and *Drosophila*, both for 'all' uORFs (present in reference species for genus) and 'conserved' uORFs (present in all species in genus; Table 2). This suggests that for some genes, selection may be operating in parallel on synonymous codon usage and uORFs to reduce translational efficiency, and that suppression of translation efficiency may be a more important mechanism of eukaryotic gene regulation than currently appreciated.

**Table 2:** Association between K_p_/K_u _*p *values and uORFs.

	*P *value for K_p_/K_u _association w/uORFs^a^	
	
	all uORFs	conserved uORFs
*Cryptococcus*	0.047	0.0069
*Saccharomyces*	0.19	0.68
*Drosophila*	0.017	0.05

^a ^Analyses performed using Mann-Whitney *U *test on FET 1-tailed *p *values

### Functional enrichment analysis

In all three genera, we identified the Gene Ontology Biological Processes that were significantly enriched for low K_p_/K_u _ratios using a Mann-Whitney *U *test (Table 3). All categories that were significantly enriched for low K_p_/K_u _ratios, and presumably inefficient translation, were either explicitly or potentially associated with regulatory or signal transduction roles.

**Table 3:** Gene Ontology Biological Processes significantly enriched for genes exhibiting low K_p_/K_u _ratios.

		Bonferroni-corrected
	GO Biological Function Category	*P *value
*Cryptococcus*	GO:0030528 Transcription regulator activity	0.00052
	GO:0084672 Protein kinase activity	0.05
*Saccharomyces*	GO:0003677 DNA-binding	0.00079
	GO:0030528 Transcription regulator activity	0.0088
	GO:0084672 Protein kinase activity	0.024
*Drosophila*	GO:0000166 Nucleotide binding	0.003
	GO:0004871 Signal Transducer Activity	0.02

## Discussion

We report strong evidence of natural selection for unpreferred codon usage across dozens of genes in *Cryptococcus *and *Drosophila*. We find an association between the simple K_p_/K_u _selection metric and translational efficiency in *Saccharomyces*, as well as a significant association between K_p_/K_u _and uORFs in *Cryptococcus *and *Drosophila*, suggesting selection is acting on translational efficiency as opposed to accuracy. Further, we report an enrichment of this selection signal in genes regulating transcription or cellular processes in all three genera. Selection for unpreferred codon usage, and potentially reduced translational efficiency, is likely to be more common than previously thought among Eukaryotes, and is a factor that must be considered in extrapolating ultimate gene expression levels from the vast amounts of transcription data now available.

Though we find that mutational bias or selection for nucleotide composition are not likely to cause the selection signal we see in *Cryptococcus *and *Drosophila*, it is possible that some non-translational factor is driving the selection pattern we observe. Recent work has shown that synonymous codon usage may be subject to constraint imposed by mRNA secondary structure[26,27], exonic splicing enhancers[28,29], and even microRNA binding[30]. Given that unpreferred codons in the lineages we examined uniformly end in an A or U nucleotide, if G/C-to-A/U mutations are more likely to induce an advantageous change in mRNA secondary structure or some other molecular phenotype than mutations in the opposite direction, such a phenotype may be the true subject of selection. These additional functional roles imposed on silent coding sites might be expected to generally reduce synonymous substitution rates, in contrast to the accelerated substitution rates that we see in selected genes, but could contribute to occasional bouts of disequilibrium in substitution patterns.

Another caveat derives from the fact that the method we use to assign preferred and unpreferred codon status assumes consistent codon preference across growth/developmental phases, tissue types, etc. Codon preferences are known to be variable within an organism (*eg *[31]) in accordance with spatially or temporally fluctuating tRNA levels. So, the possibility exists that some of the genes exhibiting accelerated unpreferred substitution in fact have inverted usage preferences and are undergoing accelerated preferred substitutions. The accelerated signals of unpreferred substitution we report do not occur within single synonymous codon families, however, but across many amino acids. For a preference inversion to generate a significant acceleration in the opposing direction (as opposed to a nonsignificant result) would thus require a consistent preference inversion across many synonymous codon families, which we consider mechanistically unlikely.

The present analysis may be considered a conservative assessment of the extent of selection for unpreferred codon usage for several additional reasons. The selection signal we report represents an average level of mutation-selection disequilibrium in codon usage across multiple species within each taxonomic group. This approach increases statistical power by boosting the observed number of synonymous substitutions per gene, but may obscure speciesspecific selection. If only one species in the group has undergone selection for unpreferred codon usage/inefficient translation, the signal of that selection will be diluted by the sister lineages that are in selection-mutation equilibrium for synonymous codon usage. Measuring K_p_/K_u _ratios for each species, as opposed to across several species at a time, yields distinct but overlapping sets of genes that show significant evidence of selection for inefficient translation (results not shown). This indicates that some of the genes we identify as having K_p_/K_u _ratios significantly less than 1 may be undergoing selection in multiple species, but also that we are overlooking some genes subject to species-specific selection. Power may also be slightly compromised in our combined-species analysis by minor divergences among species in mutation profiles or preferred/unpreferred codon assignments. Nielsen et al. [17] recently published a likelihood-based estimator of selection on codon usage that may be more suited to detailed studies of selection at loci of interest.

Despite these considerations, we found that on the order of 1% of genes in the *Cryptococcus *genome and several genes in the *Drosophila *genome exhibit a statistically significant signal of selection for unpreferred codon usage. Given that these genes are enriched for uORFs, we interpret this as selection for translational inefficiency rather than inaccuracy. There may ultimately be many reasons to select for reduced translational efficiency. Several hypotheses have recently been advanced to explain reduced translational efficiency, including facilitation the maintenance of open chromatin structure via low-level transcription [32], facilitation of domain folding during translation via reduced rates of elongation[33], as well as minimization of stochastic gene expression noise [34-36]. The expression noise hypothesis derives from recent analytical and empirical findings that low translational efficiency results in less noisy gene expression [34,36-38]. Fraser et al. [35] recently found evidence that gene expression noise may be a trait subject to natural selection, as essential genes and genes that encode subunits of protein complexes in yeast (two proposed classes of genes particularly requiring precise expression) are expressed with less predicted noise than most other genes.

The noise minimization hypothesis generates a testable prediction in the context of the current results, as analytical and empirical models show that expression noise can be propagated through gene regulatory cascades [34,38,39]. Genes in regulatory cascades that are noisily expressed might therefore engender more severe fitness consequences than noisily expressed genes in non-regulatory roles, as regulator noise may be amplified at downstream targets. We predicted, therefore, that transcriptional regulators and other classes of regulatory genes should be insulated from noise in order to prevent the propagation and amplification of expression noise through a genetic cascade.

When we looked for biological processes significantly enriched for genes exhibiting low K_p_/K_u _ratios in *Saccharomyces*, *Cryptococcus*, and *Drosophila*, we found transcriptional regulators, protein kinases, DNA/nucleotide binding genes, and proteins involved in signal transduction enriched across the three taxonomic groups (Table 3). The presence of transcriptional regulators in this list directly confirms our hypothesis. Many genes annotated as nucleotide binding are potentially regulatory as well. Protein kinases are involved in the regulation of many cellular processes, and may therefore likely command expression levels as precise as those genes regulating transcription or transducing molecular signals.

## Conclusion

Using patterns of synonymous substitution, we detect evidence of recent selection for unpreferred codon usage at dozens of genetic loci in both a fungal and insect lineage. The accelerated unpreferred synonymous substitution rates we see may result from selection for translational inefficiency or inaccuracy, or may also represent selection on mRNA secondary structure or some other molecular phenotype. This signal of positive selection is concordant with purifying selection observed at uORFs. As uORFs are another genic feature known to reduce translational efficiency, this lends credence to the hypothesis that translational inefficiency is the driver of selection, perhaps to limit expression noise. The similar functional enrichment profile observed across the fruitfly lineage and two deeply divergent fungal lineages suggests that selection to moderate expression noise of genes involved in signaling, activation, or regulation of other genes may be a general phenomenon in eukaryotic genomes, and that this noise moderation is attainable through unpreferred codon usage, uORFs, and possibly other mechanisms as yet undiscovered.

## Methods

### Sequence resources and 5' Leader/3' Trailer Mapping

We obtained the genome assemblies of four species belonging to the *Cryptococcus neoformans *species complex from the websites of the sequencing centers that produced them (strain JEC21: TIGR; strain WM276: Michael Smith Genome Center; strains H99 and R265: Broad Institute). We used gene calls from TIGR for strain JEC21. We used gene calls produced by Jason Stajich [40] for strains R265, H99, and WM276. We defined the most distal extent 5' leader sequences and 3' trailer sequences in the alignments using a library of 23,000 full-length cDNAs from strain JEC21 produced by TIGR [41]. We retained for analysis only those 5' and 3' leader/trailer sequences that showed no evidence of introns and exhibited conserved genic start/stop codons.

For *Drosophila*, we obtained the *D. melanogaster *release 4.3 assembly and annotation from FlyBase [42]. We obtained the most recent *D. simulans*, *D. sechellia*, and *D. yakuba *assemblies and annotations from the UCSC Genome Bioinformatics webpage [43]. We defined 5' leader and 3' trailer regions according to the *D. melanogaster *release 4.3 annotations.

For *Saccharomyces*, we obtained the most recent *S. cerevisiae *strain *S288C *assembly and gene calls from SGD [44]. We obtained genome assemblies for *S. paradoxus *and *S. mikatae *from the Broad Institute website [45]. We defined the most distal extent of *Saccharomyces *5' leader and 3' trailer regions using 5' SAGE data [46] as well as expression tiling array data [47]. In cases where these two data sources cited different leader lengths for the same gene, we favored the longer estimate.

### Synonymous Codon analysis

Codon analyses were performed on clusters of aligned, orthologous genes. Orthology was determined within each clade using a reciprocal-best-BLAST hit criterion implemented with a custom Perl script. Orthologs were aligned using ClustalW [48].

We evaluated codon bias for each gene in all genomes with the ENC' statistic[49]. We conferred preferred, unpreferred, and equal status on each gene after the method of Sharp and Lloyd [50], using genes that scored below the 10^th ^percentile and above the 90^th ^percentile of codon bias as 'highly' and 'lowly' biased gene sets for evaluation of relative codon usage. A heterogeneity chi square test (χ^2 ^highly biased + χ^2 ^lowly biased – χ^2 ^pooled) was used to identify divergent codon usage patterns between the two gene sets. Codons exhibiting heterogeneity χ^2 ^values greater than 7.88 (p < 0.005) among the highly and lowly biased gene sets were assigned preferred or unpreferred status; less significant usage differences were interpreted as equal status. Codon usage preferences as determined by this method are nearly identical within the *Cryptococcus*, *Drosophila*, and *Saccharomyces *genera (Additional Files 1, 2, 3). We computed tallies for each subclass of synonymous site in each gene as described in Bauer DuMont et al. [16], using an empirical substitution rate matrix derived from substitutions observed among orthologous 5' leader and 3' trailer sequences (Additional File 9). The *Drosophila *empirical rate matrix we derived using this method is very similar to that reported by Petrov and Hartl [51]. Ancestral codon states for synonymous differences observed were inferred using a maximum likelihood approach implemented in the codeml program of PAML 3.14[52]. The codon with the highest posterior probability under the marginal reconstruction approach was assumed ancestral. To avoid ambiguity, only synonymous codons differing by a single base change between their ancestral and derived states were utilized, and orthologous codons exhibiting signs of nonsynonymous change in any lineage were discarded.

We identified K_p_/K_u _ratios significantly less than one using a 1-tailed Fisher's exact test (FET). We performed false discovery rate analysis [21] on the FET *p *value distribution using the QVALUE software package[53]. This software uses a density histogram of *p *values to calculate the incidence of false positive results for *p *values less than or equal to a given value.

We analyzed the nature of the codon bias exhibited by genes showing extremely low K_p_/K_u _ratios in *Cryptococcus *using counts of different tRNAs in the *C. neoformans JEC21 *genome and the ratio of preferred to unpreferred codons in *JEC21 *genes. The number of tRNA genes per codon was calculated by counting the tRNAs identified in the official TIGR annotation of *JEC21 *and applying standard eukaryotic wobble pairing rules. The genes of *JEC21 *for which K_p_/K_u _could be calculated were then analyzed to determine the arithmetic average number of tRNAs/codon.

### uORF analysis

We conducted all analyses on uORFs with custom Perl scripts. For the purposes of this analysis we defined a uORF as an AUG triplet followed by at least one intervening codon and a stop codon (UAG, UAA, or UGA). uORFs were permitted to overlap with each other. We required uORFs to be either contained entirely within the 5' leader sequence or to overlap with the downstream coding ORF by at most a single base. We considered a uORF to be conserved if, in the multiple alignment of orthologous leader sequences, all strains exhibited a start codon and a stop codon in the same position, and those start and stop codons were in the same frame relative to each other.

### Functional enrichment analysis

We performed functional enrichment analysis for the K_p_/K_u _results using a Mann-Whitney *U *test. Gene Ontology annotations for *Drosophila melanogaster, Saccharomyces cerevisiae, and Cryptococcus neoformans *strain JEC21 were respectively obtained from FlyBase, SGD, and TIGR. Gene Ontology Biological Process categories annotated to at least 10 genes in the reference genome of each clade were used for enrichment analysis. Categories that were functionally overlapping or nested in each annotation were condensed using the GOSLIM algorithm[54] to minimize the multipletesting penalty. Speciesspecific slims were used for *Saccharomyces *and *Drosophila*; a generic slim was used for *Cryptococcus*. Bonferroni correction was applied to enrichment *p *values to compensate for the testing of multiple categories.

## Authors' contributions

DN conceived of the study, assembled the data set, carried out the analyses, and drafted the manuscript. JG assisted with guiding the analyses, interpretation of the results, and drafting of the manuscript. Both authors read and approved the final manuscript.

## Appendix 1: Rationale behind the K_p_/K_u _Statistic

That K_p_/K_u _statistic may be used to compare the relative rates of preferred and unpreferred synonymous substitution. Because an ancestral sequence may contain different numbers of preferred and preferred 'sites' (opportunities for each type of mutaiton), it is necessary to normalize the count of each class of substitution by the number of ancestral sites in each class. K_p _is defined as (no. of preferred subs/no. preferred ancestral sites), and K_u _is likewise defined as (no. of unpreferred subs/no. of unpreferred ancestral sites). Ancestral codons that are already in a preferred state tend to exhibit a higher ratio of unpreferred to preferred sites, just as unpreferred ancestral codons exhibit a higher ratio of preferred to unpreferred sites.

As an example, consider a gene encoding only one type of amino acid, tyrosine, which is encoded by only two possible synonymous codons, TAT and TAC. Further, let us assume that TAC is a preferred codon, TAT is an unpreferred codon, and that a Jukes-Cantor model of substitution applies.

We can then calculate that each TAC codon exhibits 1/3 of an unpreferred synonymous site [0 (pos. 1) + 0 (pos. 2) + 1/3 (pos. 3)], and 0 preferred synonymous sites. Likewise, TAT codons exhibit 1/3 of a preferred site, and 0 unpreferred sites. See methods section for further detail on estimating counts of sites.

If the hypothetical gene ancestrally contains 30 TAC codons and 30 TAT codons, then it exhibits (30 * 1/3 + 30 * 0) = 10 unpreferred sites and (30 * 0 + 30 * 1/3) = 10 preferred sites. That is, the ancestral sequence offers equal opportunities for both preferred and unpreferred synonymous substitutions to occur in descendant lineages.

Under conditions without selection for codon usage, and assuming no nonsynonymous mutations are tolerated, one would therefore expect on average to observe roughly equal numbers of preferred and unpreferred substitutions in descendant lineages. If 5 TAC codons turn into TAT codons, that would constitute 5 unpreferred substitutions, and K_u _would be 5/10 = 0.5. If 5 TAT codons also turn into TAC codons, K_p _is similarly 5/10 = 0.5, so the K_p_/K_u _ratio would be equal to 1 on average.

If, however, there is selection for increased unpreferred codon usage, then one might be more likely to see mutations that change TAC codons into TAT rather than vice versa. If 8 TAC-to-TAT changes and only 2 TAT-to-TAC changes occur, K_u _would be 8/10 = 0.8 and K_p _would be 2/10 = 0.2, yielding a K_p_/K_u _ratio less than 1 (0.25). Selection for greater preferred codon usage would similarly yield a K_p_/K_u _ratio that is greater than 1.

Fisher's exact test, or similar statistical tests, may be used to identify significant deviations from the equilibrium expectation that K_p_/K_u _= 1. Note that K_p_/K_u _is independent of ancestral codon usage bias, so that genes exhibiting either ancestrally high or low codon usage bias may both be expected to yield K_p_/K_u _ratios close to 1 if there is no change in selection for synonymous codon usage in any descendant lineages.

## Supplementary Material

Additional file 1 *Synonymous codon class assignments in Cryptococcus spp*. (a table of preferred, unpreferred, and equal codon assignments) Click here for file

Additional file 2 *Synonymous codon class assignments in Drosophila spp*. (a table of preferred, unpreferred, and equal codon assignments) Click here for file

Additional file 3 *Synonymous codon class assignments in Saccharomyces spp*. (a table of preferred, unpreferred, and equal codon assignments) Click here for file

Additional file 4 *Cryptococcus genes exhibiting K*_p_*/K*_u _<*1 at p *<*0.01 *(a list of genes potentially exhibiting significantly accelerated unpreferred substitution) Click here for file

Additional file 5 *Drosophila genes exhibiting K*_p_*/K*_u _<*1 at p < 0.01 *(a list of genes potentially exhibiting significantly accelerated unpreferred substitution) Click here for file

Additional file 6 *Saccharomyces genes exhibiting K*_p_*/K*_u _<*1 at p < 0.01 *(a list of genes potentially exhibiting significantly accelerated unpreferred substitution) Click here for file

Additional file 7 *Translational efficiency vs. K*_p_*/K*_u _*in Saccharomyces cerevisiae *(a graph comparing empirical measurements of translational efficiency with K_p_/K_u_) Click here for file

Additional file 8 *Lists of genes with uORFs in Cryptococcus, Drosophila, and Saccharomyces *(lists of uORFs occurring in reference species and uORFs conserved in each genus) Click here for file

Additional file 9 *Mutation profiles derived from 5' leader and 3' flanking regions *(a table of rooted mutations observed in genic flanking regions of all 3 genera) Click here for file
