# Supplementary material for: Positive selection for unpreferred codon usage in eukaryotic genomes
Source: BMC Evol Biol. 2007 Jul 18;7:119. doi: 10.1186/1471-2148-7-119 (PMC1936986; doi:10.1186/1471-2148-7-119)
Supplement: Additional file 1 — Synonymous codon class assignments in Cryptococcus spp. (a table of preferred, unpreferred, and equal codon assignments) [file 1471-2148-7-119-S1.pdf]

Additional File 1. Synonymous codon class assignments in *Cryptococcus spp.*

AA	Codon	status*	^{2}	AA	Codon	status*	^{2}
Phe	UUU	u	685.0	Ala	GCU	p	862.1
	UUC	p	685.0		GCC	p	2075.3
Leu	UUA	u	797.1		GCA	u	2319.6
	UUG	p	13.2		GCG	u	715.9
	CUU	p	840.5	Tyr	UAU	u	1192.3
	CUC	p	3047.0		UAC	p	1192.3
	CUA	u	607.2	His	CAU	u	1480.6
	CUG	u	1221.5		CAC	p	1480.6
Ile	AUU	p**	18.3	Gln	CAA	u	580.4
	AUC	p	1224.0		CAG	p	580.4
	AUA	u	1542.0	Asn	AAU	u	1918.6
Met	AUG	n/a	n/a		AAC	p	1918.6
Val	GUU	p	370.7	Lys	AAA	u	3517.0
	GUC	p	1922.0		AAG	p	3517.0
	GUA	u	988.9	Asp	GAU	u	1528.5
	GUG	u	1001.6		GAC	p	1528.5
Ser	UCU	p	2527.4	Glu	GAA	u	1462.7
	UCC	p	971.7		GAG	p	1462.7
	UCA	u	1163.4	Cys	UGU	e	5.6
	UCG	u	845.8		UGC	e	5.6
	AGU	u	228.8	Trp	UGG	n/a	n/a
	AGC	u	9.8	Arg	CGU	p	28.3
Pro	CCU	p	697.9		CGC	u	611.9
	CCC	p	1651.0		CGA	p	2060.9
	CCA	u	1562.1		CGG	u	674.9
	CCG	u	757.8		AGA	u	670.1
Thr	ACU	p	629.0		AGG	p	670.1
	ACC	p	1962.7	Gly	GGU	p	4868.0
	ACA	u	1553.6		GGC	p	16.6
	ACG	u	898.0		GGA	u	1324.4
					GGG	u	1402.5

* p = preferred codon; u = unpreferred codon; e = equal codon

** codon assigned equal status in strain *H99*
