# Supplementary material for: Positive selection for unpreferred codon usage in eukaryotic genomes
Source: BMC Evol Biol. 2007 Jul 18;7:119. doi: 10.1186/1471-2148-7-119 (PMC1936986; doi:10.1186/1471-2148-7-119)
Supplement: Additional file 2 — Synonymous codon class assignments in Drosophila spp. (a table of preferred, unpreferred, and equal codon assignments) [file 1471-2148-7-119-S2.pdf]

Additional File 2. Synonymous codon class assignments in *Drosophila spp.*

**AA**	**Codon**	**status***	χ^2	**AA**	**Codon**	**status***	χ^2
Phe	UUU	u	7189.2	Ala	GCU	u	573.4
	UUC	p	7189.2		GCC	p	11724.1
Leu	UUA	u	2833.4		GCA	u	4006.1
	UUG	u	1283.9		GCG	u	442.6
	CUU	u	2095.3	Tyr	UAU	u	3234.9
	CUC	p	286.5		UAC	p	3234.9
	CUA	u	1868.1	His	CAU	u	1313.6
	CUG	p	25963.3		CAC	p	1313.6
Ile	AUU	u	1146.5	Gln	CAA	u	5385.2
	AUC	p	10418.4		CAG	p	5385.2
	AUA	u	4652.7	Asn	AAU	u	4600.4
Met	AUG	n/a	n/a		AAC	p	4600.4
Val	GUU	u	2102.8	Lys	AAA	u	12019.1
	GUC	p	852.8		AAG	p	12019.1
	GUA	u	2391.4	Asp	GAU	u	1274.0
	GUG	p	4297.4		GAC	p	1274.0
Ser	UCU	u	744.6	Glu	GAA	u	8697.3
	UCC	p	3197.8		GAG	p	8697.3
	UCA	u	1708.8	Cys	UGU	u	1758.7
	UCG	p	1005.4		UGC	p	1758.7
	AGU	u	1147.9	Trp	UGG	n/a	n/a
	AGC	p	203.0	Arg	CGU	p**	129.6
Pro	CCU	u	793.3		CGC	p	6067.9
	CCC	p	3573.1		CGA	u	3359.9
	CCA	u	1068.2		CGG	u	980.7
	CCG	e***	1.2		AGA	u	571.7
Thr	ACU	u	1013.6		AGG	p	571.7
	ACC	p	6217.3	Gly	GGU	u	25.2
	ACA	u	1492.6		GGC	p	5098.3
	ACG	u***	70.2		GGA	u	1415.0
					GGG	u	827.7

* p = preferred codon; u = unpreferred codon; e = equal codon

** codon assigned equal status in *D. simulans* and *D. sechellia*

*** codon assigned preferred status in *D. simulans* and *D. sechellia*
