# Supplementary material for: Positive selection for unpreferred codon usage in eukaryotic genomes
Source: BMC Evol Biol. 2007 Jul 18;7:119. doi: 10.1186/1471-2148-7-119 (PMC1936986; doi:10.1186/1471-2148-7-119)
Supplement: Additional file 3 — Synonymous codon class assignments in Saccharomyces spp. (a table of preferred, unpreferred, and equal codon assignments) [file 1471-2148-7-119-S3.pdf]

Additional File 3. Synonymous codon class assignments in *Saccharomyces spp.*

AA	Codon	status*	χ^2	AA	Codon	status*	χ^2
Phe	UUU	u	728.4	Ala	GCU	p	2953.3
	UUC	p	728.4		GCC	p	75.5
Leu	UUA	u	124.2		GCA	u	1795.0
	UUG	p	4017.0		GCG	u	427.2
	CUU	u	289.4	Tyr	UAU	u	730.2
	CUC	u	87.1		UAC	p	730.2
	CUA	u	72.7	His	CAU	u	319.7
	CUG	u	301.6		CAC	p	319.7
Ile	AUU	p	19.0	Gln	CAA	p	568.3
	AUC	p	727.6		CAG	u	568.3
	AUA	u	981.5	Asn	AAU	u	1237.0
Met	AUG	n/a	n/a		AAC	p	1237.0
Val	GUU	p	762.6	Lys	AAA	u	2220.5
	GUC	p	850.7		AAG	p	2220.5
	GUA	u	945.2	Asp	GAU	u	363.0
	GUG	u	677.9		GAC	p	363.0
Ser	UCU	p	1580.2	Glu	GAA	p	693.1
	UCC	p	674.6		GAG	u	693.1
	UCA	u	463.3	Cys	UGU	p	114.0
	UCG	u	253.3		UGC	u	114.0
	AGU	u	266.6	Trp	UGG	n/a	n/a
	AGC	u	143.0	Arg	CGU	p	16.4
Pro	CCU	u	255.2		CGC	u	1365.3
	CCC	u	283.2		CGA	u	1739.0
	CCA	p	2291.9		CGG	u	1465.5
	CCG	u	227.0		AGA	p	2844.8
Thr	ACU	p	479.8		AGG	u	889.1
	ACC	p	784.5	Gly	GGU	p	5172.7
	ACA	u	907.8		GGC	u	427.6
	ACG	u	391.4		GGA	u	1001.0
					GGG	u	384.4

* p = preferred codon; u = unpreferred codon; e = equal codon
