# Supplementary material for: Positive selection for unpreferred codon usage in eukaryotic genomes
Source: BMC Evol Biol. 2007 Jul 18;7:119. doi: 10.1186/1471-2148-7-119 (PMC1936986; doi:10.1186/1471-2148-7-119)
Supplement: Additional file 4 — Cryptococcus genes exhibiting Kp/Ku <1 at p <0.01 (a list of genes potentially exhibiting significantly accelerated unpreferred substitution) [file 1471-2148-7-119-S4.pdf]

Additional File 4. *Cryptococcus* genes exhibiting $K_p/K_u < 1$ at $p < 0.01$ (Fisher's exact test)

gene	pref_subs	pref_sites	unpref_subs	unpref_sites	Kp	Ku	Kp/Ku	1-tailed p value
CNF02120	10	86.82	34	94.35	0.13	0.49	0.25	7.31E-05
CNA07150	20	142.98	41	120.89	0.15	0.45	0.34	8.39E-05
CNE01810	15	103.23	44	118.11	0.16	0.51	0.31	1.00E-04
CNK02390	0	29.63	12	33.29	0.00	0.49	0.00	1.64E-04
CND03960	32	231.58	68	251.00	0.15	0.34	0.45	1.69E-04
CNC05310	30	142.52	44	105.74	0.25	0.61	0.41	2.58E-04
CND06010	44	289.44	113	432.02	0.17	0.32	0.53	2.85E-04
CNG02100	36	176.85	68	185.30	0.24	0.50	0.47	3.19E-04
CNJ00300	19	97.08	48	114.90	0.23	0.61	0.37	3.46E-04
CNB02140	24	138.34	46	128.84	0.20	0.48	0.41	3.48E-04
CNG02300	22	151.49	44	143.99	0.16	0.39	0.41	5.25E-04
CNK01140	7	68.40	57	193.66	0.11	0.37	0.29	5.74E-04
CNK02970	45	268.86	84	294.03	0.19	0.36	0.53	6.04E-04
CNB00900	18	102.04	79	222.83	0.20	0.48	0.42	6.26E-04
CNL05700	34	158.21	54	138.37	0.25	0.55	0.46	7.27E-04
CNB04100	15	102.08	49	150.40	0.16	0.43	0.38	8.66E-04
CNC01140	11	70.63	31	80.90	0.17	0.54	0.33	9.93E-04
CNB02860	6	58.93	25	75.31	0.11	0.44	0.25	1.10E-03
CNN00810	26	163.69	66	225.63	0.18	0.37	0.48	1.10E-03
CNK00710	48	246.68	94	304.73	0.23	0.40	0.57	1.13E-03
CNL04130	19	121.01	42	127.20	0.18	0.44	0.40	1.13E-03
CND05200	37	185.72	78	234.91	0.23	0.44	0.53	1.15E-03
CNC06370	8	62.61	46	138.93	0.14	0.44	0.32	1.18E-03
CNG01650	14	118.68	44	165.95	0.13	0.33	0.39	1.25E-03
CNF03930	5	55.28	34	114.93	0.10	0.38	0.26	1.26E-03
CNA05300	31	187.26	68	230.05	0.19	0.38	0.50	1.29E-03
CNB05050	5	71.87	17	62.81	0.07	0.34	0.22	1.30E-03
CNE00660	16	116.40	39	128.33	0.15	0.39	0.39	1.38E-03
CNC00470	8	53.91	26	64.81	0.17	0.57	0.29	1.45E-03
CNN01220	45	255.53	81	284.64	0.20	0.36	0.56	1.47E-03
CNA03490	6	61.46	20	60.31	0.10	0.44	0.24	1.49E-03
CNC01280	14	96.80	35	109.36	0.16	0.42	0.39	1.92E-03
CNE00110	21	123.54	36	106.85	0.19	0.45	0.43	1.94E-03
CND05830	13	116.02	35	134.04	0.12	0.32	0.38	2.07E-03
CND04030	14	124.38	32	124.66	0.12	0.31	0.39	2.08E-03
CNB05310	20	111.24	40	114.91	0.21	0.47	0.44	2.12E-03
CNN00470	13	87.37	26	73.43	0.17	0.48	0.35	2.15E-03
CNL04400	21	121.58	48	144.24	0.20	0.44	0.45	2.25E-03
CNI02960	11	98.25	32	117.01	0.12	0.34	0.36	2.40E-03
CNG03670	39	227.08	90	324.88	0.20	0.35	0.56	2.40E-03
CNA01910	16	101.62	48	152.45	0.18	0.41	0.43	2.45E-03
CNK02350	15	87.81	46	133.39	0.19	0.46	0.42	2.50E-03
CNA00030	26	145.02	43	129.04	0.20	0.44	0.46	2.58E-03
CNH02490	38	148.53	65	157.14	0.31	0.60	0.52	2.62E-03
CNH02670	5	49.58	15	42.52	0.11	0.48	0.23	2.74E-03

CNF02560	11	92.80	28	98.27	0.13	0.36	0.36	2.89E-03
CNA05830	6	59.53	29	97.18	0.11	0.38	0.28	2.90E-03
CNG01380	7	39.74	18	37.66	0.20	0.76	0.26	2.93E-03
CNK01570	18	115.01	30	91.85	0.18	0.43	0.41	2.98E-03
CNC05100	1	25.36	12	33.26	0.04	0.49	0.08	2.99E-03
CND05480	14	104.28	34	115.65	0.15	0.37	0.40	3.04E-03
CNC05730	18	130.90	52	195.83	0.15	0.33	0.46	3.05E-03
CNC05090	29	199.77	56	220.57	0.16	0.31	0.52	3.10E-03
CNA07530	11	79.92	42	139.45	0.15	0.39	0.39	3.60E-03
CNH02850	32	163.11	67	206.45	0.23	0.43	0.53	3.69E-03
CNA07940	8	61.62	23	68.50	0.14	0.45	0.32	3.79E-03
CNJ01740	6	61.23	32	115.62	0.11	0.35	0.30	3.82E-03
CNL04410	4	35.81	22	57.18	0.12	0.54	0.22	3.92E-03
CNN01490	19	130.85	51	187.39	0.16	0.34	0.48	3.94E-03
CNK00670	16	82.80	28	71.71	0.22	0.55	0.41	3.95E-03
CNC00480	20	94.51	31	77.77	0.25	0.57	0.44	4.00E-03
CNB02720	10	72.40	18	51.83	0.15	0.47	0.33	4.06E-03
CNE01260	6	45.57	26	70.22	0.14	0.51	0.28	4.19E-03
CNC03120	32	214.11	79	321.49	0.17	0.30	0.56	4.36E-03
CND05770	1	41.05	11	49.40	0.02	0.26	0.09	4.70E-03
CNK00930	14	128.51	32	137.92	0.12	0.28	0.42	4.73E-03
CNA03280	23	156.46	40	148.59	0.16	0.33	0.49	4.83E-03
CNH02920	3	24.30	15	31.53	0.13	0.75	0.18	4.84E-03
CNL05940	1	22.11	17	51.98	0.05	0.43	0.11	4.86E-03
CNI03390	37	202.50	64	220.55	0.21	0.37	0.57	4.89E-03
CNL05530	6	37.71	21	47.11	0.18	0.68	0.26	4.91E-03
CNC01340	11	98.98	28	109.56	0.12	0.31	0.38	4.92E-03
CNA06750	38	194.85	73	240.28	0.23	0.39	0.58	4.95E-03
CNE01370	15	68.97	33	78.50	0.26	0.62	0.42	4.96E-03
CNN00530	30	174.11	51	173.01	0.20	0.37	0.52	4.99E-03
CNC02650	28	158.44	48	159.60	0.20	0.38	0.52	5.04E-03
CND06150	27	129.15	81	238.83	0.25	0.45	0.54	5.47E-03
CND04680	26	179.41	46	178.57	0.16	0.32	0.51	5.50E-03
CNH01860	42	201.21	83	265.87	0.24	0.40	0.61	5.59E-03
CNF04310	1	36.85	10	41.34	0.03	0.29	0.09	5.59E-03
CNG01170	12	71.59	26	72.55	0.19	0.49	0.39	5.61E-03
CNI02520	4	31.64	11	24.49	0.14	0.69	0.20	5.66E-03
CNK00890	17	107.64	35	112.35	0.18	0.40	0.44	5.73E-03
CNB01970	30	197.23	69	274.25	0.17	0.31	0.55	5.73E-03
CNG03400	12	64.36	27	67.16	0.21	0.58	0.37	5.80E-03
CNM01840	11	78.93	26	84.00	0.15	0.40	0.39	5.82E-03
CNN00640	14	87.16	29	86.51	0.18	0.44	0.41	5.85E-03
CNN00960	5	43.78	16	45.91	0.12	0.47	0.26	6.00E-03
CNK00730	18	98.06	40	116.86	0.21	0.46	0.46	6.04E-03
CNF02730	9	67.39	23	72.77	0.15	0.41	0.36	6.08E-03
CNI03350	16	104.28	32	106.80	0.17	0.38	0.45	6.27E-03
CNA07550	51	225.60	97	299.83	0.27	0.42	0.64	6.30E-03
CNB00070	1	18.12	11	27.95	0.06	0.56	0.10	6.48E-03
CNM02300	3	34.99	14	41.44	0.09	0.45	0.20	6.54E-03
CNA04500	11	61.60	23	59.97	0.20	0.54	0.38	6.65E-03

CNJ00810	36	202.71	58	206.50	0.20	0.35	0.58	6.89E-03
CND05270	1	23.34	17	56.97	0.04	0.38	0.12	6.94E-03
CNC05030	1	24.54	11	33.37	0.04	0.43	0.10	7.07E-03
CNC04910	5	69.59	18	80.98	0.08	0.26	0.29	7.10E-03
CNA05850	4	48.02	23	85.82	0.09	0.33	0.27	7.18E-03
CNF02900	9	73.69	22	75.93	0.13	0.37	0.36	7.22E-03
CNM01790	22	122.29	51	166.91	0.21	0.39	0.52	7.36E-03
CNL03980	14	68.69	28	70.39	0.24	0.57	0.42	7.39E-03
CNL06360	6	39.90	12	27.55	0.17	0.65	0.26	7.48E-03
CNA03050	23	127.54	46	149.82	0.21	0.39	0.52	7.71E-03
CNH01870	1	30.16	12	47.94	0.03	0.30	0.11	7.75E-03
CNF04020	23	144.62	46	164.27	0.18	0.35	0.51	7.84E-03
CNM01890	11	67.84	29	85.97	0.18	0.45	0.41	7.87E-03
CNB00770	17	97.11	32	95.01	0.20	0.45	0.45	7.93E-03
CNC02500	26	154.22	39	132.66	0.19	0.37	0.51	8.08E-03
CNH02020	9	52.26	27	73.00	0.20	0.51	0.38	8.13E-03
CNE01100	18	99.93	40	123.39	0.21	0.42	0.49	8.30E-03
CNL04710	15	105.96	40	148.15	0.16	0.33	0.47	8.45E-03
CNI02370	10	60.50	38	109.25	0.19	0.47	0.40	8.64E-03
CNJ00760	5	46.53	16	50.93	0.12	0.41	0.28	8.68E-03
CNB02270	21	152.25	31	119.53	0.15	0.32	0.48	8.74E-03
CNC04500	9	56.59	26	74.94	0.18	0.47	0.38	8.77E-03
CNJ01760	23	119.57	36	108.95	0.22	0.44	0.51	8.97E-03
CNK00560	34	185.76	68	241.52	0.21	0.35	0.59	9.08E-03
CNB01740	12	83.15	56	199.23	0.16	0.35	0.46	9.20E-03
CNA06970	23	114.18	58	173.68	0.23	0.44	0.53	9.39E-03
CNB04470	1	20.45	21	67.81	0.05	0.40	0.13	9.45E-03
CNB00030	5	52.58	25	95.97	0.10	0.32	0.32	9.47E-03
CNA01290	20	119.36	64	223.62	0.19	0.36	0.53	9.63E-03
CNB03820	2	23.43	11	28.73	0.09	0.54	0.17	9.72E-03
