# Supplementary material for: Positive selection for unpreferred codon usage in eukaryotic genomes
Source: BMC Evol Biol. 2007 Jul 18;7:119. doi: 10.1186/1471-2148-7-119 (PMC1936986; doi:10.1186/1471-2148-7-119)
Supplement: Additional file 5 — Drosophila genes exhibiting Kp/Ku <1 at p < 0.01 (a list of genes potentially exhibiting significantly accelerated unpreferred substitution) [file 1471-2148-7-119-S5.pdf]

Additional File 5. *Drosophila* genes exhibiting $K_p/K_u < 1$ at $p < 0.01$ (Fisher's exact test)

gene	pref_subs	pref_sites	unpref_subs	unpref_sites	Kp	Ku	Kp/Ku	1-tailed p value
CG6690-PA	4	121.03	29	155.86	0.03	0.21	0.16	4.03E-05
CG6539-PA	10	208.86	44	274.77	0.05	0.18	0.27	4.64E-05
CG18095-PA	7	121.36	35	166.52	0.06	0.25	0.24	1.60E-04
CG33288-PB	7	223.74	41	358.13	0.03	0.12	0.26	1.74E-04
CG3491-PA	13	316.83	54	486.19	0.04	0.12	0.35	2.11E-04
CG6630-PA	39	463.27	99	645.85	0.09	0.17	0.52	2.85E-04
CG31622-PA	0	79.66	13	103.14	0.00	0.14	0.00	4.30E-04
CG9506-PA	10	157.59	92	558.46	0.07	0.19	0.36	4.46E-04
CG14376-PA	12	167.72	45	241.94	0.08	0.21	0.35	5.07E-04
CG17046-PA	12	364.55	79	953.16	0.03	0.09	0.38	5.57E-04
CG32121-PA	6	92.07	62	299.76	0.07	0.24	0.28	6.73E-04
CG1487-PA	1	90.03	18	141.76	0.01	0.14	0.08	7.57E-04
CG4903-PA	23	453.63	66	633.19	0.05	0.11	0.47	8.87E-04
CG16965-PA	8	134.92	38	217.67	0.06	0.20	0.31	9.21E-04
CG15326-PA	6	94.00	45	221.88	0.07	0.24	0.28	9.34E-04
CG32238-PA	8	110.37	36	169.47	0.08	0.25	0.31	1.02E-03
CG9626-PA	17	320.24	76	666.44	0.06	0.12	0.45	1.07E-03
CG5394-PA	23	325.80	93	685.57	0.07	0.15	0.50	1.13E-03
CG32026-PA	9	165.44	33	210.26	0.06	0.18	0.32	1.15E-03
CG7628-PA	2	91.42	37	287.56	0.02	0.14	0.16	1.24E-03
CG3327-PC	3	151.31	41	450.78	0.02	0.10	0.21	1.26E-03
CG8833-PA	12	188.09	47	300.79	0.07	0.18	0.38	1.28E-03
CG30084-PF	8	234.70	58	609.59	0.03	0.10	0.34	1.28E-03
CG7803-PA	2	77.90	37	251.84	0.03	0.16	0.16	1.33E-03
CG4063-PA	5	131.07	37	280.79	0.04	0.14	0.27	1.67E-03
CG7341-PB	1	74.64	16	118.64	0.01	0.15	0.09	1.91E-03
CG7526-PB	29	344.00	71	466.84	0.09	0.17	0.53	2.04E-03
CG5374-PB	1	68.18	40	312.81	0.01	0.14	0.11	2.08E-03
CG16742-PA	21	367.50	41	342.94	0.06	0.13	0.46	2.11E-03
CG5849-PA	11	188.26	48	343.97	0.06	0.15	0.39	2.18E-03
CG2189-PA	2	95.74	27	232.31	0.02	0.13	0.17	2.54E-03
CG10422-PA	1	66.51	20	146.07	0.02	0.15	0.10	2.93E-03
CG13840-PA	1	70.63	23	184.15	0.01	0.14	0.10	3.19E-03
CG11856-PA	34	548.61	90	864.83	0.06	0.11	0.58	3.40E-03
CG18368-PA	0	254.96	7	211.92	0.00	0.03	0.00	3.69E-03
CG31304-PA	12	237.43	68	620.74	0.05	0.12	0.44	3.74E-03
CG7223-PB	13	119.77	67	302.96	0.12	0.26	0.45	3.75E-03
CG6490-PA	19	250.98	74	519.41	0.08	0.16	0.51	3.99E-03
CG11259-PA	4	261.90	16	253.49	0.02	0.07	0.23	4.03E-03
CG14157-PA	8	89.93	28	118.02	0.09	0.29	0.33	4.06E-03
CG1677-PA	12	155.90	63	385.66	0.08	0.18	0.44	4.24E-03
CG4881-PB	11	228.41	56	516.39	0.05	0.12	0.42	4.44E-03
CG31805-PA	5	110.18	16	98.15	0.05	0.18	0.25	4.48E-03
CG7012-PB	9	125.36	46	267.06	0.08	0.20	0.39	4.52E-03
CG5747-PA	27	352.25	84	634.33	0.08	0.15	0.55	4.58E-03
CG8961-PA	1	134.45	11	152.19	0.01	0.08	0.10	5.10E-03

CG8540-PA	5	111.25	17	109.61	0.05	0.17	0.27	5.25E-03
CG33128-PA	0	35.95	33	226.96	0.00	0.16	0.00	5.35E-03
CG8102-PA	4	96.91	24	166.32	0.04	0.16	0.26	5.45E-03
CG31510-PA	13	290.99	27	260.04	0.05	0.11	0.41	5.65E-03
CG13295-PA	5	85.40	35	200.17	0.06	0.20	0.31	5.77E-03
CG7003-PA	15	225.59	59	449.95	0.07	0.14	0.48	5.90E-03
CG5149-PA	7	92.46	31	158.79	0.08	0.23	0.35	6.09E-03
CG12785-PA	16	257.28	50	409.82	0.06	0.13	0.49	6.55E-03
CG3809-PA	3	66.61	27	159.49	0.05	0.19	0.24	6.57E-03
CG18609-PA	3	48.85	23	98.04	0.06	0.28	0.23	7.19E-03
CG11762-PA	1	56.87	15	106.28	0.02	0.16	0.11	7.21E-03
CG8147-PA	2	83.07	26	226.56	0.02	0.12	0.20	7.49E-03
CG3810-PB	8	162.34	47	399.02	0.05	0.13	0.40	7.75E-03
CG9117-PA	0	36.85	13	86.06	0.00	0.17	0.00	7.92E-03
CG5455-PC	10	144.60	39	252.39	0.07	0.17	0.42	8.40E-03
CG18405-PA	3	140.08	32	406.95	0.02	0.08	0.26	8.63E-03
CG4866-PA	0	26.71	15	80.43	0.00	0.21	0.00	8.80E-03
CG14882-PA	6	81.95	32	167.12	0.08	0.22	0.35	8.87E-03
CG11349-PA	14	144.95	34	174.20	0.10	0.23	0.46	8.99E-03
CG18112-PA	2	54.40	36	223.60	0.04	0.18	0.21	9.30E-03
CG1523-PA	3	81.44	38	290.46	0.04	0.14	0.26	9.31E-03
CG4694-PA	3	79.60	24	170.10	0.04	0.16	0.25	9.38E-03
CG3616-PA	3	61.80	39	235.94	0.05	0.19	0.27	9.83E-03
CNI03390	37	202.50	64	220.55	0.21	0.37	0.57	4.89E-03
CNL05530	6	37.71	21	47.11	0.18	0.68	0.26	4.91E-03
CNC01340	11	98.98	28	109.56	0.12	0.31	0.38	4.92E-03
CNA06750	38	194.85	73	240.28	0.23	0.39	0.58	4.95E-03
CNE01370	15	68.97	33	78.50	0.26	0.62	0.42	4.96E-03
CNN00530	30	174.11	51	173.01	0.20	0.37	0.52	4.99E-03
CNC02650	28	158.44	48	159.60	0.20	0.38	0.52	5.04E-03
CND06150	27	129.15	81	238.83	0.25	0.45	0.54	5.47E-03
CND04680	26	179.41	46	178.57	0.16	0.32	0.51	5.50E-03
CNH01860	42	201.21	83	265.87	0.24	0.40	0.61	5.59E-03
CNF04310	1	36.85	10	41.34	0.03	0.29	0.09	5.59E-03
CNG01170	12	71.59	26	72.55	0.19	0.49	0.39	5.61E-03
CNI02520	4	31.64	11	24.49	0.14	0.69	0.20	5.66E-03
CNK00890	17	107.64	35	112.35	0.18	0.40	0.44	5.73E-03
CNB01970	30	197.23	69	274.25	0.17	0.31	0.55	5.73E-03
CNG03400	12	64.36	27	67.16	0.21	0.58	0.37	5.80E-03
CNM01840	11	78.93	26	84.00	0.15	0.40	0.39	5.82E-03
CNN00640	14	87.16	29	86.51	0.18	0.44	0.41	5.85E-03
CNN00960	5	43.78	16	45.91	0.12	0.47	0.26	6.00E-03
CNK00730	18	98.06	40	116.86	0.21	0.46	0.46	6.04E-03
CNF02730	9	67.39	23	72.77	0.15	0.41	0.36	6.08E-03
CNI03350	16	104.28	32	106.80	0.17	0.38	0.45	6.27E-03
CNA07550	51	225.60	97	299.83	0.27	0.42	0.64	6.30E-03
CNB00070	1	18.12	11	27.95	0.06	0.56	0.10	6.48E-03
CNM02300	3	34.99	14	41.44	0.09	0.45	0.20	6.54E-03
CNA04500	11	61.60	23	59.97	0.20	0.54	0.38	6.65E-03
CNJ00810	36	202.71	58	206.50	0.20	0.35	0.58	6.89E-03

CND05270	1	23.34	17	56.97	0.04	0.38	0.12	6.94E-03
CNC05030	1	24.54	11	33.37	0.04	0.43	0.10	7.07E-03
CNC04910	5	69.59	18	80.98	0.08	0.26	0.29	7.10E-03
CNA05850	4	48.02	23	85.82	0.09	0.33	0.27	7.18E-03
CNF02900	9	73.69	22	75.93	0.13	0.37	0.36	7.22E-03
CNM01790	22	122.29	51	166.91	0.21	0.39	0.52	7.36E-03
CNL03980	14	68.69	28	70.39	0.24	0.57	0.42	7.39E-03
CNL06360	6	39.90	12	27.55	0.17	0.65	0.26	7.48E-03
CNA03050	23	127.54	46	149.82	0.21	0.39	0.52	7.71E-03
CNH01870	1	30.16	12	47.94	0.03	0.30	0.11	7.75E-03
CNF04020	23	144.62	46	164.27	0.18	0.35	0.51	7.84E-03
CNM01890	11	67.84	29	85.97	0.18	0.45	0.41	7.87E-03
CNB00770	17	97.11	32	95.01	0.20	0.45	0.45	7.93E-03
CNC02500	26	154.22	39	132.66	0.19	0.37	0.51	8.08E-03
CNH02020	9	52.26	27	73.00	0.20	0.51	0.38	8.13E-03
CNE01100	18	99.93	40	123.39	0.21	0.42	0.49	8.30E-03
CNL04710	15	105.96	40	148.15	0.16	0.33	0.47	8.45E-03
CNI02370	10	60.50	38	109.25	0.19	0.47	0.40	8.64E-03
CNJ00760	5	46.53	16	50.93	0.12	0.41	0.28	8.68E-03
CNB02270	21	152.25	31	119.53	0.15	0.32	0.48	8.74E-03
CNC04500	9	56.59	26	74.94	0.18	0.47	0.38	8.77E-03
CNJ01760	23	119.57	36	108.95	0.22	0.44	0.51	8.97E-03
CNK00560	34	185.76	68	241.52	0.21	0.35	0.59	9.08E-03
CNB01740	12	83.15	56	199.23	0.16	0.35	0.46	9.20E-03
CNA06970	23	114.18	58	173.68	0.23	0.44	0.53	9.39E-03
CNB04470	1	20.45	21	67.81	0.05	0.40	0.13	9.45E-03
CNB00030	5	52.58	25	95.97	0.10	0.32	0.32	9.47E-03
CNA01290	20	119.36	64	223.62	0.19	0.36	0.53	9.63E-03
CNB03820	2	23.43	11	28.73	0.09	0.54	0.17	9.72E-03
