# Supplementary material for: Positive selection for unpreferred codon usage in eukaryotic genomes
Source: BMC Evol Biol. 2007 Jul 18;7:119. doi: 10.1186/1471-2148-7-119 (PMC1936986; doi:10.1186/1471-2148-7-119)
Supplement: Additional file 6 — Saccharomyces genes exhibiting Kp/Ku <1 at p < 0.01 (a list of genes potentially exhibiting significantly accelerated unpreferred substitution) [file 1471-2148-7-119-S6.pdf]

Additional File 6. *Saccharomyces* genes exhibiting $K_p/K_u < 1$ at $p < 0.01$ (Fisher's exact test)

gene	pref_subs	pref_sites	unpref_subs	unpref_sites	Kp	Ku	Kp/Ku	1-tailed p value
TOR2	155	595.32	163	673.05	0.32	0.29	1.09	0.00E+00
HOP1	25	140.74	48	134.86	0.20	0.48	0.42	4.37E-04
FRE1	7	133.56	28	152.52	0.05	0.21	0.26	4.41E-04
YMC1	5	60.86	29	96.83	0.09	0.38	0.23	6.33E-04
BOS1	1	37.06	13	43.86	0.03	0.38	0.07	9.49E-04
APC1	67	384.26	99	374.89	0.20	0.33	0.61	1.37E-03
PEX28	17	131.08	38	133.34	0.14	0.36	0.40	1.38E-03
MUM2	12	94.05	28	88.79	0.14	0.41	0.34	1.62E-03
GLN3	19	158.45	42	167.54	0.13	0.31	0.43	1.79E-03
TFC3	49	273.11	69	240.15	0.21	0.36	0.57	2.59E-03
AME1	10	64.16	22	55.39	0.17	0.57	0.31	2.60E-03
YMR171C	26	135.40	45	131.71	0.22	0.46	0.49	2.94E-03
YOR129C	7	172.37	20	157.21	0.04	0.14	0.30	3.58E-03
TOS3	16	124.72	30	110.46	0.14	0.34	0.42	3.73E-03
PCL6	13	95.97	28	93.39	0.15	0.38	0.39	4.03E-03
YLR446W	17	97.04	35	100.63	0.20	0.47	0.43	4.18E-03
YAP3	5	50.81	16	49.45	0.11	0.42	0.25	4.33E-03
PNT1	17	96.01	32	90.34	0.20	0.48	0.42	4.60E-03
BUR6	2	29.75	13	36.03	0.07	0.49	0.14	5.08E-03
NHA1	17	197.32	41	235.58	0.09	0.20	0.46	5.10E-03
RNH1	12	77.41	27	78.23	0.17	0.46	0.38	5.17E-03
KRE29	16	100.28	33	102.68	0.18	0.42	0.43	5.19E-03
GTR2	21	95.56	30	72.23	0.26	0.61	0.43	5.48E-03
MSC2	29	164.55	47	155.00	0.20	0.39	0.52	5.83E-03
YKU70	32	158.53	47	139.29	0.24	0.45	0.52	6.07E-03
BIT2	23	138.58	35	114.20	0.19	0.39	0.48	6.53E-03
YGR122W	9	80.37	23	83.55	0.12	0.34	0.35	6.69E-03
YAL037W	3	55.95	11	45.04	0.06	0.30	0.19	7.09E-03
YPR013C	8	64.52	22	72.67	0.14	0.39	0.35	7.19E-03
LRG1	41	242.85	65	250.76	0.19	0.32	0.60	7.57E-03
RMP1	3	45.58	11	39.25	0.07	0.35	0.20	8.83E-03
PET100	3	35.50	9	25.54	0.09	0.48	0.19	8.88E-03
FRE6	8	135.79	21	138.28	0.06	0.17	0.36	9.07E-03
PHM6	1	20.54	9	23.33	0.05	0.54	0.09	9.12E-03
YGR237C	35	177.58	57	187.92	0.23	0.39	0.59	9.49E-03
NPL6	11	111.64	23	104.76	0.11	0.26	0.41	9.65E-03
