# Supplementary material for: Positive selection for unpreferred codon usage in eukaryotic genomes
Source: BMC Evol Biol. 2007 Jul 18;7:119. doi: 10.1186/1471-2148-7-119 (PMC1936986; doi:10.1186/1471-2148-7-119)
Supplement: Additional file 7 — Translational efficiency vs. Kp/Ku in Saccharomyces cerevisiae (a graph comparing empirical measurements of translational efficiency with Kp/Ku) [file 1471-2148-7-119-S7.pdf]

Additional File 7

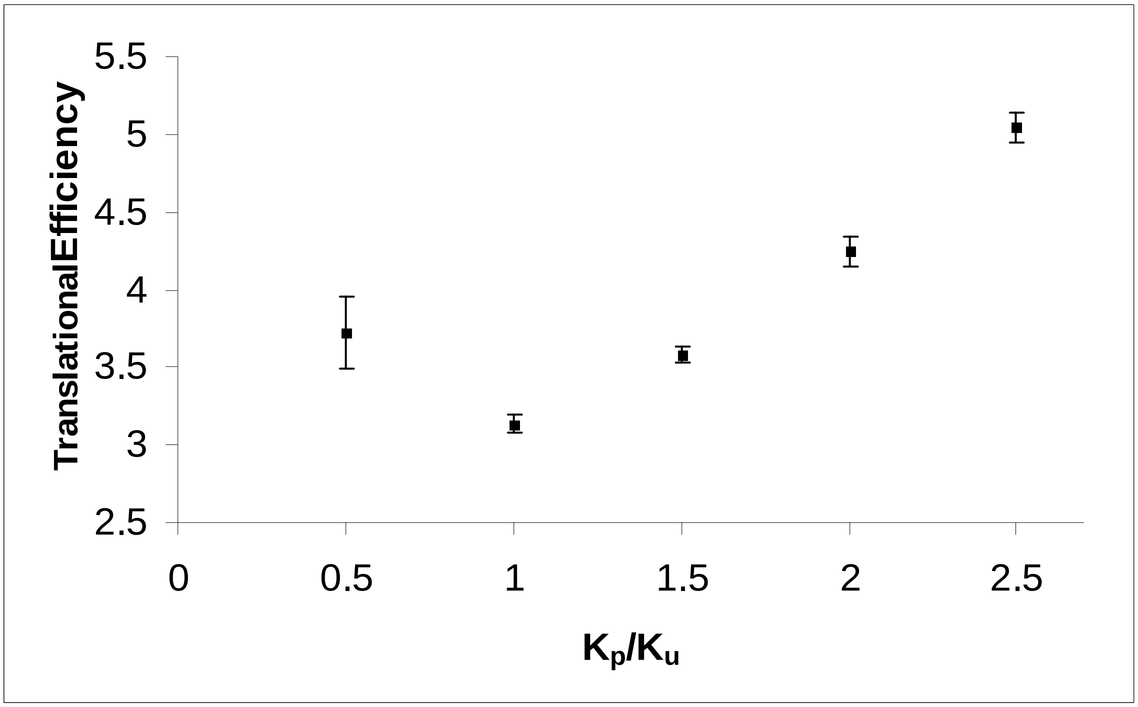

Translational efficiency is significantly correlated with Kp/Ku in Saccharomyces cerevisiae (Spearman's rho = 0.029; $p < 0.00001$). Translational efficiency values were derived from empirical data[1].

1: MacKay VL, Li X, Flory MR, Turcott E, Law GL, Serikawa KA, Xu XL, Lee H, Goodlett DR, Aebersold R, Zhao LP, Morris DR.
Gene expression analyzed by high-resolution state array analysis and quantitative proteomics: response of yeast to mating pheromone.
Mol Cell Proteomics. 2004 May;3(5):478-89. Epub 2004 Feb 6.
PMID: 14766929 [PubMed - indexed for MEDLINE]
