# Supplementary material for: Positive selection for unpreferred codon usage in eukaryotic genomes
Source: BMC Evol Biol. 2007 Jul 18;7:119. doi: 10.1186/1471-2148-7-119 (PMC1936986; doi:10.1186/1471-2148-7-119)
Supplement: Additional file 8 — Lists of genes with uORFs in Cryptococcus, Drosophila, and Saccharomyces (lists of uORFs occurring in reference species and uORFs conserved in each genus) [file 1471-2148-7-119-S8.pdf]

Additional File 8

Cryptococcus genes with at least one uORF in reference strain (JEC21):

CNA00030	CNA00210	CNA00400	CNA00630	CNA00680
CNA00690	CNA00880	CNA01140	CNA01280	CNA01590
CNA01610	CNA01930	CNA02060	CNA02150	CNA02610
CNA02720	CNA03080	CNA03150	CNA03850	CNA04130
CNA04250	CNA04290	CNA04660	CNA04690	CNA04760
CNA05050	CNA05330	CNA05410	CNA05590	CNA05920
CNA06050	CNA06310	CNA06520	CNA06530	CNA06610
CNA07240	CNA07430	CNA07560	CNA07940	CNA08100
CNA08190	CNB00170	CNB00520	CNB00650	CNB00850
CNB00980	CNB01850	CNB01890	CNB02080	CNB02410
CNB02460	CNB02580	CNB02730	CNB03300	CNB03670
CNB04030	CNB04110	CNB04180	CNB04340	CNB05480
CNB05530	CNB05710	CNB05740	CNB05750	CND01310
CND01330	CND01380	CND01430	CND01900	CND02100
CND02150	CND02270	CND02460	CND02670	CND02770
CND03090	CND03160	CND03310	CND03400	CND03640
CND04080	CND04140	CND04500	CND04620	CND04710
CND04750	CND04870	CND05280	CND05330	CND05600
CND05620	CND06170	CND06180	CNE00500	CNE01300
CNE01500	CNE01570	CNE01580	CNE02070	CNE02240
CNE02570	CNE02700	CNE03010	CNE03570	CNE03600
CNE03660	CNE03970	CNE03980	CNE04120	CNE04130
CNE04180	CNE04480	CNE04770	CNE04840	CNE04970
CNF01210	CNF01280	CNF01580	CNF01760	CNF01940
CNF02030	CNF02110	CNF02350	CNF02390	CNF02560
CNF02680	CNF02810	CNF02850	CNF02930	CNF02940
CNF02980	CNF03240	CNF03260	CNF03750	CNF03960
CNF04090	CNF04220	CNG00120	CNG00390	CNG00440
CNG00640	CNG00990	CNG01040	CNG01410	CNG01530
CNG01550	CNG01590	CNG01690	CNG01970	CNG02190
CNG02270	CNG02430	CNG02520	CNG03170	CNG03330
CNG03580	CNG03930	CNG03960	CNG04110	CNG04280
CNG04310	CNG04660	CNH03770	CNH03300	CNH03100
CNH02880	CNH02120	CNH02060	CNH01670	CNH01650
CNH01190	CNH00600	CNH00380	CNH00290	CNI00090
CNI00380	CNI00420	CNI00740	CNI01370	CNI01670
CNI01780	CNI01870	CNI01890	CNI02760	CNI02980
CNI03200	CNI03420	CNI03450	CNI04160	CNJ00300
CNJ00320	CNJ00430	CNJ00600	CNJ00870	CNJ00900
CNJ01090	CNJ01190	CNJ01600	CNJ01610	CNJ01700
CNJ01860	CNJ02580	CNJ02620	CNJ02710	CNJ03150
CNJ03190	CNK00130	CNK00140	CNK00770	CNK00780

CNK00880	CNK00890	CNK00920	CNK01200	CNK01340
CNK01510	CNK01680	CNK01690	CNK01850	CNK02050
CNK02320	CNK02420	CNK02690	CNK02740	CNK02850
CNK03450	CNL03770	CNL03880	CNL04370	CNL04400
CNL04520	CNL04770	CNL05080	CNL05290	CNL05710
CNL05850	CNL05890	CNL05970	CNL06000	CNL06020
CNL06100	CNL06340	CNL06680	CNM00260	CNM00660
CNM00790	CNM00900	CNM01640	CNM01880	CNM01960
CNM02370	CNM02500	CNN00530	CNN00790	CNN00970
CNN01660				

Cryptococcus genes with at least one **conserved** uORF:

CNA00030	CNA00210	CNA00630	CNA00880	CNA01140
CNA01590	CNA01610	CNA01930	CNA02060	CNA02150
CNA03080	CNA03150	CNA04690	CNA05050	CNA05330
CNA05410	CNA05920	CNA07240	CNA07560	CNA08190
CNB00170	CNB00520	CNB01850	CNB01890	CNB02080
CNB02460	CNB03300	CNB04180	CNB04340	CNB05530
CNB05740	CND01310	CND01380	CND02100	CND02270
CND02460	CND02670	CND03400	CND04500	CND05330
CND05620	CND06180	CNE00500	CNE01300	CNE02070
CNE03660	CNE03970	CNE04120	CNE04130	CNE04180
CNE04480	CNE04970	CNF02030	CNF02110	CNF02930
CNF02940	CNF03260	CNF03750	CNF03960	CNG00440
CNG01040	CNG01410	CNG01550	CNG02190	CNG02270
CNG02430	CNG03580	CNG04110	CNG04280	CNG04660
CNH03770	CNH02880	CNH02120	CNH02060	CNH01190
CNH00380	CNI00420	CNI01670	CNI01780	CNI02760
CNI03450	CNI04160	CNJ01700	CNJ01860	CNJ02620
CNJ02710	CNK00780	CNK00920	CNK01200	CNK01510
CNK01690	CNK01850	CNK02320	CNK02420	CNK03450
CNL03770	CNL03880	CNL04370	CNL04400	CNL04520
CNL04770	CNL06000	CNM00260	CNM00660	CNM00790
CNM00900	CNN00530			

Drosophila genes with at least one uORF in reference species (*melanogaster*):

CG11357-PA	CG4840-PA	CG13213-PC	CG7979-PA	CG7187-PC
CG9027-PB	CG2503-PA	CG9134-PB	CG16987-PA	CG4433-PB
CG5344-PB	CG31284-PC	CG3073-PA	CG10687-PA	CG8600-PA
CG10639-PA	CG3613-PA	CG1079-PA	CG4218-PA	CG4019-PA
CG9072-PA	CG10660-PA	CG10869-PA	CG13890-PA	CG4670-PA
CG2224-PA	CG9643-PA	CG8815-PD	CG17054-PD	CG12797-PA
CG17377-PC	CG4068-PB	CG3771-PA	CG32308-PB	CG7075-PA
CG12210-PB	CG17717-PA	CG8323-PA	CG8080-PA	CG2813-PA
CG10653-PA	CG5378-PA	CG1883-PD	CG4707-PA	CG7499-PA
CG8776-PA	CG11926-PA	CG9772-PA	CG30001-PA	CG3502-PA
CG8152-PA	CG6870-PA	CG6199-PB	CG4585-PA	CG1009-PE
CG9045-PE	CG8091-PA	CG2993-PA	CG33713-PA	CG4659-PA
CG10757-PA	CG2244-PB	CG8372-PB	CG18336-PA	CG2046-PA
CG9165-PA	CG16983-PE	CG17019-PA	CG12283-PA	CG9326-PB
CG32037-PA	CG10754-PA	CG14657-PB	CG7740-PC	CG8604-PA
CG10949-PA	CG2023-PA	CG13949-PA	CG17248-PE	CG31961-PB
CG8444-PA	CG7142-PA	CG12317-PB	CG4356-PA	CG9175-PB
CG31365-PA	CG3637-PA	CG7182-PA	CG3476-PA	CG10522-PA
CG5973-PC	CG5215-PB	CG7020-PA	CG12073-PA	CG18397-PA
CG32491-PN	CG8300-PA	CG10160-PA	CG11887-PA	CG18330-PA
CG12750-PA	CG6190-PA	CG14992-PA	CG17603-PC	CG31694-PA
CG13366-PA	CG5146-PA	CG4427-PA	CG2718-PC	CG1133-PA
CG4681-PA	CG10221-PA	CG8290-PB	CG13475-PA	CG8657-PA
CG8309-PA	CG4622-PA	CG2616-PA	CG3798-PF	CG33472-PA
CG6549-PC	CG12289-PA	CG9582-PA	CG12162-PA	CG8298-PA
CG1868-PB	CG4370-PA	CG18317-PA	CG11063-PB	CG6282-PB
CG12090-PC	CG7910-PA	CG6249-PA	CG5824-PA	CG8785-PA
CG13784-PA	CG3935-PA	CG13123-PA	CG9536-PA	CG4063-PA
CG11523-PA	CG17258-PA	CG8039-PA	CG9358-PA	CG2916-PB
CG10173-PA	CG13907-PA	CG11421-PA	CG18314-PC	CG5387-PA
CG31908-PA	CG6015-PA	CG9044-PA	CG9263-PA	CG18210-PA
CG10742-PA	CG18642-PA	CG4677-PB	CG1309-PA	CG9181-PA
CG3860-PA	CG31259-PA	CG8121-PB	CG9921-PA	CG7870-PA
CG4813-PA	CG18003-PB	CG10072-PA	CG3929-PA	CG5026-PB
CG10217-PB	CG4496-PA	CG3658-PA	CG13345-PA	CG6191-PA
CG3977-PA	CG9114-PA	CG33650-PA	CG8368-PA	CG2934-PA
CG4379-PA	CG4213-PA	CG12404-PA	CG4944-PB	CG15309-PA
CG12740-PB	CG2128-PA	CG7492-PA	CG15580-PB	CG8830-PB
CG14939-PA	CG10337-PA	CG8814-PA	CG7125-PA	CG3165-PA
CG15822-PC	CG7035-PB	CG8312-PA	CG9224-PA	CG7201-PA
CG2846-PA	CG16758-PF	CG7830-PA	CG30427-PA	CG7927-PA
CG10574-PA	CG5354-PA	CG13933-PA	CG15880-PA	CG31786-PA
CG8230-PA	CG8005-PA	CG7745-PA	CG7261-PA	CG5888-PA

CG5846-PA	CG3436-PA	CG1024-PA	CG13920-PA	CG8704-PA
CG11907-PB	CG31546-PA	CG9657-PA	CG6315-PA	CG6376-PB
CG6619-PA	CG6412-PA	CG5837-PA	CG11883-PB	CG6121-PA
CG4527-PC	CG12730-PA	CG17509-PA	CG1492-PA	CG6353-PA
CG7524-PF	CG31974-PA	CG7986-PB	CG11753-PA	CG2108-PA
CG8525-PA	CG32280-PC	CG8493-PB	CG9104-PA	CG17611-PA
CG11737-PA	CG12030-PA	CG6705-PB	CG9242-PA	CG3955-PA
CG6816-PA	CG33505-PA	CG11576-PA	CG11660-PA	CG6674-PA
CG12016-PB	CG3702-PA	CG7161-PA	CG6329-PB	CG8520-PA
CG6321-PA	CG12772-PA	CG7115-PB	CG8816-PA	CG5012-PA
CG7164-PA	CG3075-PA	CG4042-PA	CG4623-PA	CG12902-PB
CG10904-PA	CG2943-PA	CG9948-PA	CG9222-PA	CG7149-PA
CG9014-PA	CG10898-PA	CG1962-PC	CG3183-PA	CG9828-PB
CG3727-PC	CG11679-PA	CG12316-PA	CG12052-PQ	CG4058-PA
CG10528-PA	CG1418-PA	CG15386-PA	CG4079-PA	CG17124-PA
CG8631-PA	CG10198-PA	CG9172-PA	CG3194-PA	CG1444-PA
CG32407-PA	CG12831-PA	CG17285-PA	CG8839-PE	CG2031-PA
CG10212-PA	CG6627-PA	CG3810-PB	CG11990-PA	CG6704-PA
CG6988-PA	CG12025-PA	CG7109-PA	CG10703-PA	CG10541-PA
CG10121-PD	CG4449-PA	CG12026-PA	CG6181-PB	CG9281-PB
CG14762-PA	CG18869-PA	CG12342-PA	CG4230-PA	CG3654-PD
CG9323-PA	CG8236-PA	CG5263-PB	CG32381-PA	CG7044-PA
CG14655-PA	CG8556-PA	CG7197-PA	CG1318-PD	CG10254-PB
CG4775-PA	CG9780-PA	CG4323-PB	CG6575-PA	CG1134-PA
CG10060-PA	CG3587-PA	CG10206-PA	CG12891-PA	CG10372-PA
CG9523-PA	CG8116-PA	CG9008-PC	CG3504-PA	CG33162-PA
CG5295-PA	CG12846-PA	CG8529-PE	CG12249-PA	CG3567-PA
CG13222-PA	CG12084-PA	CG33097-PA	CG5087-PA	CG3876-PA
CG4237-PA	CG5823-PA	CG7104-PA	CG1137-PA	CG12189-PA
CG10462-PA	CG7516-PA	CG32372-PA	CG1101-PA	CG8616-PA
CG17608-PA	CG11206-PA	CG8560-PA	CG12186-PA	CG30415-PA
CG3309-PA	CG4852-PA	CG4609-PA	CG12391-PA	CG3790-PA
CG33691-PB	CG6543-PA	CG7223-PB	CG17723-PE	CG32022-PA
CG7047-PA	CG7004-PB	CG32343-PA	CG9258-PA	CG31635-PA
CG8409-PA	CG31805-PA	CG5001-PA	CG9302-PA	CG12127-PA
CG13148-PB	CG1172-PA	CG14971-PA	CG12341-PA	CG10252-PA
CG3409-PA	CG10467-PA	CG12069-PA	CG9147-PA	CG14440-PA
CG1381-PA	CG4690-PA	CG7112-PA	CG12099-PB	CG10144-PA
CG16932-PB	CG1107-PB	CG31176-PA	CG2928-PA	CG14299-PA
CG5171-PA	CG13893-PA	CG13322-PA	CG4545-PA	CG8277-PA
CG4445-PA	CG2852-PA	CG2221-PA	CG5371-PA	CG2677-PA
CG11440-PA	CG4468-PA	CG7300-PB	CG6903-PA	CG9153-PA
CG2161-PB	CG10776-PA	CG3488-PA	CG7915-PA	CG3556-PA
CG15251-PA	CG5261-PA	CG1146-PA	CG12091-PA	CG13305-PA
CG3595-PA	CG3335-PA	CG14968-PB	CG1935-PA	CG6845-PA
CG4114-PA	CG13326-PA	CG13400-PA	CG4692-PB	CG5893-PA

CG8833-PA	CG4389-PA	CG7503-PA	CG7948-PA	CG30362-PA
CG5486-PB	CG17084-PA	CG14636-PA	CG8563-PA	CG2938-PB
CG9261-PD	CG7154-PA	CG10385-PA	CG3797-PA	CG10576-PA
CG3331-PA	CG1898-PA	CG9159-PA	CG6757-PA	CG12075-PB
CG6767-PA	CG7479-PA	CG6097-PA	CG8516-PA	CG3403-PA
CG30365-PA	CG6647-PA	CG11188-PA	CG6207-PC	CG4141-PB
CG4914-PA	CG9211-PA	CG2872-PB	CG3539-PC	CG17184-PB
CG13601-PA	CG32700-PB	CG9663-PA	CG12187-PA	CG12470-PA
CG6709-PA	CG3644-PB	CG13398-PA	CG6297-PB	CG1599-PA
CG8772-PB	CG9586-PB	CG15862-PB	CG7009-PA	CG10253-PA
CG15147-PA	CG3501-PA	CG17068-PA	CG5651-PA	CG7359-PA
CG5687-PA	CG4422-PA	CG10480-PA	CG7576-PA	CG9267-PA
CG10539-PA	CG18802-PA	CG9135-PB	CG30431-PA	CG4241-PC
CG5288-PC	CG2841-PC	CG2818-PA	CG1515-PA	CG7175-PA
CG13211-PB	CG6713-PB	CG12240-PA	CG2845-PA	CG16717-PA
CG33261-PE	CG11567-PA	CG3735-PA	CG12155-PA	CG14351-PA
CG12141-PA	CG7108-PA	CG11455-PB	CG2922-PG	CG12393-PA
CG7764-PA	CG15013-PB	CG2911-PA	CG3279-PA	CG11508-PA
CG13098-PA	CG3074-PB	CG30023-PA	CG5273-PB	CG31839-PA
CG6513-PA	CG10973-PA	CG6230-PA	CG10988-PA	CG7018-PA
CG9779-PA	CG5638-PA	CG8553-PB	CG7379-PA	CG7772-PA
CG1317-PB	CG2051-PB	CG10830-PA	CG9515-PA	CG8596-PA
CG12159-PA	CG3127-PA	CG7912-PA	CG13253-PA	CG8057-PA
CG7042-PA	CG1071-PA	CG17181-PA	CG7200-PA	CG10387-PA
CG12410-PA	CG10103-PA	CG5906-PA	CG5383-PA	CG3159-PA
CG32300-PB	CG4675-PB	CG1488-PB	CG2086-PB	CG2127-PA
CG4217-PB	CG8024-PB	CG6016-PB	CG18171-PA	CG2097-PA
CG17712-PA	CG7222-PA	CG9717-PA	CG5392-PA	CG11201-PA
CG14030-PA	CG9035-PA	CG31849-PA	CG3192-PA	CG4643-PA
CG11309-PB	CG10596-PB	CG1900-PA	CG8032-PA	CG4454-PA
CG2698-PA	CG8357-PA	CG3022-PA	CG2114-PA	CG15154-PA
CG9853-PA	CG7737-PA	CG14825-PA	CG31689-PB	CG5337-PA
CG1034-PD	CG6583-PA	CG4293-PA	CG6416-PF	CG4057-PA
CG3925-PA	CG10691-PB	CG12935-PA	CG11427-PA	CG1049-PC
CG8597-PE	CG32103-PB	CG14536-PA	CG10808-PA	CG33489-PA
CG3078-PA	CG10590-PA	CG10908-PA	CG10698-PA	CG14804-PA
CG7803-PA	CG7962-PA	CG8964-PA	CG3249-PA	CG3835-PA
CG6743-PA	CG2857-PA	CG5461-PA	CG9100-PC	CG11760-PB
CG1004-PA	CG2216-PD	CG17030-PA	CG10947-PC	CG5339-PA
CG11444-PA				

Drosophila genes with at least one **conserved** uORF:

CG4840-PA	CG13213-PC	CG7979-PA	CG7187-PC	CG9027-PB
CG2503-PA	CG9134-PB	CG16987-PA	CG4433-PB	CG5344-PB
CG3073-PA	CG10687-PA	CG10639-PA	CG3613-PA	CG1079-PA
CG4218-PA	CG10660-PA	CG13890-PA	CG4670-PA	CG2224-PA
CG8815-PD	CG12797-PA	CG4068-PB	CG3771-PA	CG7075-PA
CG17717-PA	CG8323-PA	CG8080-PA	CG5378-PA	CG1883-PD
CG4707-PA	CG7499-PA	CG8776-PA	CG11926-PA	CG8152-PA
CG6870-PA	CG4585-PA	CG9045-PE	CG2993-PA	CG33713-PA
CG4659-PA	CG2244-PB	CG2046-PA	CG9165-PA	CG17019-PA
CG12283-PA	CG9326-PB	CG14657-PB	CG7740-PC	CG2023-PA
CG17248-PE	CG31961-PB	CG8444-PA	CG7142-PA	CG12317-PB
CG4356-PA	CG9175-PB	CG31365-PA	CG3637-PA	CG3476-PA
CG10522-PA	CG7020-PA	CG12073-PA	CG18397-PA	CG8300-PA
CG11887-PA	CG18330-PA	CG6190-PA	CG31694-PA	CG13366-PA
CG5146-PA	CG4427-PA	CG2718-PC	CG1133-PA	CG4681-PA
CG10221-PA	CG8657-PA	CG4622-PA	CG2616-PA	CG3798-PF
CG33472-PA	CG6549-PC	CG12162-PA	CG4370-PA	CG11063-PB
CG6282-PB	CG12090-PC	CG7910-PA	CG5824-PA	CG8785-PA
CG13784-PA	CG3935-PA	CG13123-PA	CG9536-PA	CG4063-PA
CG11523-PA	CG8039-PA	CG13907-PA	CG18314-PC	CG5387-PA
CG31908-PA	CG6015-PA	CG9044-PA	CG18642-PA	CG4677-PB
CG1309-PA	CG3860-PA	CG31259-PA	CG7870-PA	CG18003-PB
CG3929-PA	CG5026-PB	CG10217-PB	CG4496-PA	CG3658-PA
CG6191-PA	CG3977-PA	CG9114-PA	CG33650-PA	CG8368-PA
CG2934-PA	CG4379-PA	CG12404-PA	CG12740-PB	CG2128-PA
CG7492-PA	CG8830-PB	CG14939-PA	CG8814-PA	CG15822-PC
CG7035-PB	CG8312-PA	CG7201-PA	CG16758-PF	CG7830-PA
CG30427-PA	CG7927-PA	CG10574-PA	CG13933-PA	CG15880-PA
CG31786-PA	CG8230-PA	CG8005-PA	CG7745-PA	CG7261-PA
CG5846-PA	CG3436-PA	CG13920-PA	CG6315-PA	CG6619-PA
CG6412-PA	CG5837-PA	CG6121-PA	CG4527-PC	CG12730-PA
CG17509-PA	CG1492-PA	CG7524-PF	CG7986-PB	CG11753-PA
CG2108-PA	CG8525-PA	CG8493-PB	CG12030-PA	CG6705-PB
CG9242-PA	CG3955-PA	CG6816-PA	CG33505-PA	CG11576-PA
CG11660-PA	CG6674-PA	CG3702-PA	CG7161-PA	CG6329-PB
CG8520-PA	CG6321-PA	CG7115-PB	CG5012-PA	CG7164-PA
CG4042-PA	CG12902-PB	CG10904-PA	CG2943-PA	CG9948-PA
CG9222-PA	CG7149-PA	CG10898-PA	CG1962-PC	CG3183-PA
CG9828-PB	CG3727-PC	CG11679-PA	CG12316-PA	CG1418-PA
CG15386-PA	CG17124-PA	CG10198-PA	CG9172-PA	CG3194-PA
CG32407-PA	CG12831-PA	CG8839-PE	CG2031-PA	CG6627-PA
CG3810-PB	CG11990-PA	CG12025-PA	CG10703-PA	CG10541-PA
CG10121-PD	CG12342-PA	CG3654-PD	CG5263-PB	CG32381-PA

CG7044-PA	CG14655-PA	CG8556-PA	CG1318-PD	CG10254-PB
CG4775-PA	CG4323-PB	CG6575-PA	CG1134-PA	CG10060-PA
CG10206-PA	CG10372-PA	CG8116-PA	CG9008-PC	CG3504-PA
CG33162-PA	CG12249-PA	CG3567-PA	CG13222-PA	CG3876-PA
CG4237-PA	CG7104-PA	CG7516-PA	CG1101-PA	CG17608-PA
CG11206-PA	CG30415-PA	CG3309-PA	CG4852-PA	CG3790-PA
CG33691-PB	CG7223-PB	CG17723-PE	CG7047-PA	CG7004-PB
CG32343-PA	CG9258-PA	CG31635-PA	CG5001-PA	CG9302-PA
CG13148-PB	CG14971-PA	CG12341-PA	CG3409-PA	CG14440-PA
CG1381-PA	CG7112-PA	CG12099-PB	CG10144-PA	CG16932-PB
CG1107-PB	CG31176-PA	CG2928-PA	CG14299-PA	CG13893-PA
CG13322-PA	CG4545-PA	CG8277-PA	CG4445-PA	CG2852-PA
CG11440-PA	CG4468-PA	CG7300-PB	CG6903-PA	CG9153-PA
CG2161-PB	CG10776-PA	CG3488-PA	CG1146-PA	CG12091-PA
CG13305-PA	CG3595-PA	CG3335-PA	CG14968-PB	CG4114-PA
CG13400-PA	CG4692-PB	CG5893-PA	CG4389-PA	CG7948-PA
CG30362-PA	CG17084-PA	CG8563-PA	CG2938-PB	CG7154-PA
CG10385-PA	CG3797-PA	CG3331-PA	CG1898-PA	CG6757-PA
CG12075-PB	CG7479-PA	CG6097-PA	CG8516-PA	CG3403-PA
CG6647-PA	CG11188-PA	CG6207-PC	CG4141-PB	CG4914-PA
CG9211-PA	CG2872-PB	CG3539-PC	CG32700-PB	CG9663-PA
CG3644-PB	CG6297-PB	CG8772-PB	CG15862-PB	CG15147-PA
CG3501-PA	CG17068-PA	CG7359-PA	CG5687-PA	CG7576-PA
CG9267-PA	CG18802-PA	CG9135-PB	CG30431-PA	CG5288-PC
CG2841-PC	CG2818-PA	CG7175-PA	CG13211-PB	CG6713-PB
CG12240-PA	CG2845-PA	CG11567-PA	CG14351-PA	CG12393-PA
CG7764-PA	CG11508-PA	CG30023-PA	CG5273-PB	CG10973-PA
CG6230-PA	CG7018-PA	CG5638-PA	CG8553-PB	CG7379-PA
CG7772-PA	CG1317-PB	CG10830-PA	CG9515-PA	CG3127-PA
CG13253-PA	CG8057-PA	CG1071-PA	CG17181-PA	CG7200-PA
CG10387-PA	CG12410-PA	CG10103-PA	CG5906-PA	CG5383-PA
CG3159-PA	CG32300-PB	CG2086-PB	CG8024-PB	CG6016-PB
CG18171-PA	CG2097-PA	CG7222-PA	CG9717-PA	CG5392-PA
CG11201-PA	CG31849-PA	CG3192-PA	CG4643-PA	CG8032-PA
CG2698-PA	CG8357-PA	CG3022-PA	CG15154-PA	CG9853-PA
CG7737-PA	CG14825-PA	CG31689-PB	CG5337-PA	CG1034-PD
CG4293-PA	CG6416-PF	CG4057-PA	CG12935-PA	CG11427-PA
CG8597-PE	CG32103-PB	CG14536-PA	CG10808-PA	CG3078-PA
CG10908-PA	CG10698-PA	CG7803-PA	CG8964-PA	CG3835-PA
CG5461-PA	CG9100-PC	CG1004-PA	CG2216-PD	CG10947-PC
CG5339-PA	CG11444-PA			

Saccharomyces genes with at least one uORF in reference strain (*cerevisiae*):

YOL138C HPA2 SHU2 JAC1 YER130C RTS1 PUS2 BEM1
YML020W YCK1 LAS17 KOG1 CYC3 ODC2 PEX22 YDL025C GLG1
YSY6 NPT1 YOR004W RRP46 YMR233W MEU1 HUS1 YPR114W
YTM1 DAL7 YPR091C STE13 YLR307C-A LEU9 ZRT1 MSC3 IES3
RPS26B PPH21 ATO3 SBP1 VMA6 OST2 RBG1 YGR251W SKY1
YHL026C SGO1 LYS9 RPI1 SBH1 BUD20 YJR054W NDE1
MRM2 BSP1 SFK1 PMD1 PRP43 RPL24B YMR130W SPT15 RMD5
RCR1 RAD59 NAS2 YLR364W FAS1 LHP1 YJR096W
YGL059W YLR073C RPL18A UGA3 FYV7 MTR2 HEM12
TGS1 YGR068C KTI12 PFD1 YNG1 AQY2 YLR301W RPL35A
YGL176C MDM35 TES1 PCT1 YDR387C YOR338W
TOM40 YNR034W-A VMA21 PMU1 PAC2 YJL213W
YJR116W HOT13 MKS1 STP2 ALT2 RMT2 HFA1 POP3 YPC1
RPS24A YBR220C SSN8 YMR166C COX13 HTB1 ARV1
BUR6 MTM1 SLA1 RDS2 FEN2 SGE1 CMP2 BNI5 YNL211C LPP1
STE2 RXT3 GUT1 KRE11 PDR16 FMP50 MOD5 CFD1 MRS3
YHR138C CTS1 YCR015C PAN2 YGR149W DBF4 YPL191C
RAD24 YMR090W SRP68 RRN9 PPM1 DOA1 YBL009W RHO3
YHR112C YER087W RBA50 HSH49 UBA3 AGX1 GTR1
YEL006W RRP14 YOL057W LST7 YGL250W CIN5 RRN6
YCR023C YMC2 YPR109W SNF12 RPL30 YOL036W YTA7
YNL187W MAD2 ATP18 IPI3 YDR090C YKL098W VPS8 TMT1
RGS2 WHI5 PHO89 ARO9 SAP185 MEP2 YER139C GCN3
LYS12 BCH1 ERG7 GSP2 URA6 IDI1 YOR051C HEM3 ORT1 URK1
EAF6 SMY2 YIL083C UGA1 YMR204C YLR137W VMA13
PTC4 YOL159C-A BFR2 RGP1 SIT4 DCC1 YIL055C APP1 UBR2
HAP4 ACC1 IST2 PHM8 RPC11 SGT1 PUF2 MFA1 YBR028C GAD1
NDC1 VHT1 DIN7 YKL050C YHR159W RPS22A APA2 RSC6
BYE1 RPP1B SPT2 YIM1 EBS1 RIB5 YIR003W YET2 ARP6
CDC36 CHS5 PCL5 NTF2 RPL32 MET3 ECM7 YKL151C RAD1
NOP10 AGA2 YKR022C MET32 SER3 YDL146W ADE8
PDA1 ELC1 RCN1 YOR352W MRP51 GCN4 MRPL31 PEP1
VPS35 MKK2 YPL225W YFR045W PTC7 ADA2 THG1 GTT1 NVJ1
YHR009C SRB8 SEM1 BAP3 RHO1 FET4 TIF34 YLR168C
YBR108W RTG2 YOR304C-A SUR1 ARP7 TBF1 YBL028C
YBL029C-A POP1 YGL114W MCH1 SOH1 HUB1 UME6 ALG5 MNN2
IMP3 CIT1 COX4 PEX12 UMP1 YGR093W GCN5 SFH1 YOL125W
CSM2 YKR017C PGK1 RPC40 ERI1 AGP1 SRP102 OST6
YMR034C BAS1 ERG26 COX14 RDH54 SSY5 DSS4
YOL015W RBF9 RLF2 YIR035C CBP1 RIF2 YPL034W YNG2
SIA1 DLD1 YOR044W CDC21 DUR3 ISA1 SDS22 YDR128W
YGR131W YLR413W YDR539W BCK2 YMR171C FAF1 HTZ1
ATG5 RPT5 PSY3 CWC21 CWC27 MAL33 AGP2 SEC65

MSE1	DCW1	FMO1	YFR017C		DUS3	MNR2	RAD53		RRN11	
YDL176W		AFT2	STN1	BDH1	ECM2	FSH2	YBL039W-B		VPS55	RAI1
PPZ1	ALR1	YBR285W		MVD1	JIP4	ERV14		YER137C		TPK1
PET111		FIG1	TRS33	CSE1	SYP1	YNL191W		UBC8	IPK1	SPB4
HRB1	LSM1	DLD3	YAP1	PIC2	RMD6	YHL010C		YNL254C		
YAL027W		AMS1	RPF1	DBR1	YRO2	ARO4	RPB4	NRG1	CLN3	
YDR306C		PAN6	SNU66		APN1	EXO84		GID7		

Saccharomyces genes with at least one **conserved** uORF:

YER130C PUS2 YCK1 KOG1 GLG1 YPR091C ZRT1 MSC3
RPS26B SBP1 OST2 YHL026C SGO1 RPI1 BUD20
YJR054W NDE1 PMD1 RPL24B FAS1 LHP1 YLR073C YNG1
RPL35A YOR338W YJL213W MKS1 HFA1 RPS24A
YBR220C SSN8 ARV1 BUR6 MTM1 RDS2 STE2 MRS3 CTS1 SRP68
RHO3 YEL006W LST7 RRN6 YOL036W YNL187W TMT1
SAP185 BCH1 ERG7 URA6 YOR051C HEM3 URK1 UGA1 BFR2
YIL055C APP1 HAP4 ACC1 PHM8 RPC11 NDC1 RPL32 ECM7
YKL151C RAD1 YKR022C SER3 YDL146W PDA1 ELC1 GCN4
MKK2 THG1 YBR108W ARP7 YGL114W COX4 PGK1 ERI1
YMR034C COX14 RDH54 YPL034W YLR413W BCK2
ATG5 MAL33 AGP2 YFR017C MNR2 YDL176W VPS55 ALR1
JIP4 PET111 SYP1 HRB1 YRO2 NRG1 CLN3 YDR306C
