# Supplementary material for: Positive selection for unpreferred codon usage in eukaryotic genomes
Source: BMC Evol Biol. 2007 Jul 18;7:119. doi: 10.1186/1471-2148-7-119 (PMC1936986; doi:10.1186/1471-2148-7-119)
Supplement: Additional file 9 — Mutation profiles derived from 5' leader and 3' flanking regions (a table of rooted mutations observed in genic flanking regions of all 3 genera) [file 1471-2148-7-119-S9.pdf]

Additional File 9. Mutation profiles derived from 5' leader and 3' flanking regions

		relative mutation rates (scaled to sum to 1)		
from	to	*Cryptococcus*	*Saccharomyces*	*Drosophila*
A	C	0.03	0.04	0.06
A	G	0.18	0.22	0.11
A	T	0.04	0.05	0.08
T	G	0.03	0.02	0.04
T	A	0.04	0.05	0.08
T	C	0.17	0.19	0.11
C	T	0.19	0.15	0.15
C	G	0.03	0.03	0.04
C	A	0.04	0.04	0.09
G	C	0.03	0.03	0.04
G	T	0.03	0.03	0.07
G	A	0.19	0.16	0.12

Rates derived from 19,322 mutations in *Cryptococcus,*
1,074 mutations in *Saccharomyces* , and 3,776 mutations in *Drosophila*
